# Content and design considerations for digital supportive care tools for patients with cancer: A textual statistical analysis of patients’, relatives’, and healthcare professionals’ perspectives

**DOI:** 10.1371/journal.pone.0360665

**Published:** 2026-10-29

**Authors:** Louise Zanni, Martin Rebillon, Ambre Naeyaert, Valentyn Fournier, Lisa Laroussi-Libeault, Pauline Justin, Pascal Antoine, Carole Haryan, Louise Richez, Kristopher Lamore

**Affiliations:** 1 Université of Lille, CNRS, UMR - SCALab - Sciences Cognitives et Sciences Affectives, Lille, France; 2 Université Paris Cité, Laboratoire de Psychopathologie et Processus de Santé, Boulogne-Billancourt, France; 3 Maisonneuve-Rosemont Hospital Research Center, Montréal, Quebec, Canada; 4 Division of Hematology, Oncology and Transplantation, Université de Montréal, Montréal, Quebec, Canada. Department of Anesthesiology and Pain Medicine, Faculty of Medicine, Université de Montréal, Montréal, Québec, Canada; 5 Department of Anesthesiology and Pain Medicine, Faculty of Medicine, Université de Montréal, Montréal, Québec, Canada; 6 Division of Hematology, Oncology and Transplantation, Université de Montréal, Montréal, Quebec, Canada; University of the West of England, UNITED KINGDOM OF GREAT BRITAIN AND NORTHERN IRELAND

## Abstract

Access to supportive care remains a major public health concern. Digital tools are increasingly used in healthcare to address supportive care needs. It is essential to understand the needs and perspectives of the end users of these tools, including patients, relatives, and healthcare professionals (HCPs), to design and implement an option that effectively supports cancer patients and their relatives. This study was conducted to document and compare the perspectives of patients, relatives, and HCPs in France to identify perceived facilitators and barriers to the use of digital supportive care tools, as well as these individuals’ expectations regarding tool development. Semi-structured interviews were conducted with three different groups: 38 patients with cancer (including two unusable interviews), 20 relatives, and 43 HCPs. The interviews were analysed using textual statistical analysis in IRaMuTeQ. Four classes emerged from the analysis: (1) cancer experience, (2) patient care, (3) imagination and positioning in the conceptualisation of the digital tool: the content, and (4) tool shape: the container. Across the three groups, there was broad agreement regarding identified needs and potential solutions. Participants did not conceptualise digital supportive tools as a care or treatment device, but rather as an informational and reflexive space, centred on the ability to seek, think, choose, and express oneself, while preserving human mediation. This finding is important because it highlights a shift in digital health toward a customisable empowerment tool that integrates patients and relatives, accounts for usage barriers, and is designed as an optional complement rather than a prescriptive solution. The results also highlight the importance of user-centred, accessible, and tailored approaches in tool development.

## Introduction

Supportive care has played a central role in the management of patients with cancer since the early 2000s in France, with an earlier focus developing internationally, particularly in the United States. Although the definition of supportive care remains somewhat broad, it encompasses a comprehensive set of interventions that address patients’ physical, psychological, and social needs. The goals of supportive care include improving quality of life, managing treatment-related side effects, and organizing the patient’s healthcare journey from diagnosis through remission or end-of-life care. Thus, supportive care is structured around the principle of providing individualised care while empowering patients to play an active role in managing their illness [1]. Despite its importance, access to supportive care remains a major public health concern.

Indeed, disease symptoms and treatment side effects, such as fatigue [2] or cognitive impairment [3,4], can reduce patients’ ability to engage with supportive services. Moreover, healthcare professional (HCP) shortages in certain regions [5,6] further constrain access to supportive care. In France, these challenges are compounded by persistent social health inequalities, with geographic disparities playing a particularly significant role [7].

In this context, digital tools have become increasingly prevalent in healthcare [8]. Such tools comprise mobile applications, online platforms, and digital health systems designed to support patients’ care, self-management, and well-being. For example, in the context of lung cancer, a previous systematic review highlighted the positive effects of electronic health applications delivering physical activity, exercise, or training interventions on patients’ well‑being [9]. However, the review also emphasised key challenges in implementing such tools. Additionally, Fournier et al. [10] underscored that despite their potential, digital supportive care tools are unevenly adopted, with patients’ symptom burden and regional disparities in healthcare resources acting as major barriers to their uptake. One central issue in implementing digital tools is the need for HCPs to be familiar with and confident in using these solutions to effectively introduce them to patients [11]. Another potential limitation of studies assessing the impact of digital tools lies in the wide variety of available solutions (e.g., teleconsultations, mobile health applications, and remote monitoring platforms), which differ in their functionalities, levels of interactivity, and intended purposes; thus, they may not yield comparable outcomes or levels of effectiveness. Hence, both patients and HCPs may experience difficulties in navigating or adopting these tools, further complicating their integration into routine care.

Digital supportive care tools have predominantly been developed to address patient and HCP care-monitoring needs, which largely relate to symptom management, psychological functioning, and social functioning [12]. However, unmet needs extend far beyond these areas and have been categorised into 11 primary domains: physical, psychosocial/emotional, family‑related, social, interpersonal/intimacy, practical, daily living, spiritual/existential, health system/information, patient–clinician communication, and cognitive [13,14]. Nevertheless, patients are not the only individuals requiring supportive care. Relatives often assume a demanding and long-term role, and their own psychological, informational, and practical needs remain substantial [15]. Although studies have shown that insufficient supportive care for relatives significantly reduces their quality of life at all stages of the patient’s illness [16], few digital solutions are currently designed specifically for them. This gap underscores the need to develop supportive interventions that also address relatives’ experiences, ensuring a more comprehensive approach to cancer care.

Thus, to design and implement a digital tool that is truly supportive for patients with cancer in France, it is essential to understand the needs and perspectives of the end users: patients, relatives, and HCPs. This study was conducted to document and compare the speech patterns of these groups to identify facilitators and barriers to the use of digital tools intended to reduce the impact of cancer on patients’ and relatives’ quality of life, and to clarify these individuals’ expectations for such a tool.

## Methods

### Design

This qualitative, exploratory, and multicentre study was preregistered on Open Science Framework (OSF: https://doi.org/10.17605/OSF.IO/P4ME6 and https://doi.org/10.17605/OSF.IO/FZJ94). The results presented in this article are derived from a secondary, integrated analysis of interviews. The initial analysis involved a thematic analysis [17] of participants’ accounts, conducted separately for each group [18–20]. Textual statistical analyses were conducted to identify the most representative categories within the participants’ discourse. This approach was employed to capture broad, recurrent themes across the dataset, complementing the more nuanced, less frequent themes identified through thematic analysis. This method also facilitated the analysis of implicit meanings in the discourse.

Ethical approvals were obtained from the Research Ethics Committee of the University of Lille in France (Numbers 2022–640-S110 and 2023–699-S17) and comply with the General Data Protection Regulation. The study was conducted in accordance with the tenets of the 1964 Helsinki Declaration, the ethical principles of the French Code of Ethics for Psychologists, and the 2016 APA Ethical Principles of Psychologists and Code of Conduct. All participants received comprehensive information about the study’s objectives and their right to withdraw at any time, both before and during the study. As required by law and reported to the ethics committee, oral consent was obtained from all participants upon inclusion and audio-recorded before the research interview began and audio-recorded before the research interview began.

### Population

Three categories of participants were recruited: patients, relatives, and HCPs.

For inclusion, participants were required to be adults (over 18 years of age), fluent in French, and have received a cancer diagnosis, whether currently in treatment or not. Relatives were included if they were providing or had provided support to a patient with cancer or a survivor (including deceased patients). HCPs were included if they worked with patients with cancer in adult oncology departments in public hospitals or private practice in France.

Patients and relatives with current or previous psychiatric or cognitive disorders (diagnosed for patients, self-reported for relatives) were non-included, as were patients presenting a deterioration in their general health status that would prevent their participation in the study. No non-inclusion criteria were specified for HCPs.

### Procedure

Recruitment was conducted through multiple channels. The study was promoted to patients and relatives via social media (LinkedIn), patient associations (i.e., regional committee of the French National League Against Cancer, Espace Resources Cancers), and medical institutions (Comprehensive Cancer Center Oscar Lambret and University Hospital of Lille, France). Interested individuals were invited to contact VF (Ph.D., postdoctoral researcher in health psychology, male) or AN (Ph.D. candidate in health psychology, female). Upon contact, prospective participants received detailed study information by email and were asked to confirm their willingness and consent to participate. Online or face-to-face interviews were subsequently organised. Patients were given €70 as compensation for their participation, due to a funding acquired specifically for conducting this sub-group study.

For HCPs, the study was offered via an invitation email and messages on social media (LinkedIn). A list of HCPs (n = 8,000) with whom the research team was unfamiliar before recruitment working in adult oncology departments was created by searching for publicly available email addresses online. This list included medical professionals (physicians, nurses, and pharmacists) and supportive care professionals (psychologists, social workers, and dietitians). Eligible participants were contacted randomly by email, based on their workplace and profession. If they were interested, they were invited to contact AN. An information letter was subsequently sent, and upon their agreement, a videoconference interview was scheduled.

In the socio-demographic questionnaire given to patients and relatives, digital perceived competencies were assessed by asking them to rate their competence using various digital devices (e.g., computers, tablets, and phones). These data were grouped to assess their overall skill level.

Patient interviews were conducted via videoconference and/or face-to-face from the 30^th^ January 2024 to the 15^th^ April 2024 by VF or KL (Ph.D., a permanent researcher in health psychology, specializing in qualitative research, male), with relatives interviewed via videoconference between the 13^th^ February 2024 and the 15^th^ January 2025 by AN, VF, and CD (third-year undergraduate psychology student, female). HCPs were interviewed via videoconference between the 8^th^ December 2023 and the 19^th^ April 2024 by AN and JH (a Master level student in psychology, female). Interviews are based on standardized guides. First, the researcher explained what a digital health tool was, then continued with open-ended questions exploring the participants’ perceptions of psychosocial digital tools, including perceived benefits, barriers, facilitators, and needs. Various socio-demographic and medical information was gathered. At the beginning of the interview, the participants were asked to imagine a new digital tool to assist patients and their families and to share their thoughts on the subject. All the interviews were recorded and transcribed by AN, CD, CR (a Master level student in psychology, female), JH, LC (Ph.D, a research assistant in psychology, female), LR (MSc, a research assistant in psychology, female), MR (MSc, a research assistant, male), and VF. For analysis purposes, the transcripts were reworked to remove all speech marks and stage directions. Words referring to common constructs (i.e., cities, surnames, and first names) were grouped under the same names to study their occurrences.

All study materials (e.g., flyers, email content, and the interview guide) were discussed with LLL (MSc in economy, a partner patient, female) and are available on OSF.

### Data analysis

Textual statistical analyses were performed using IRaMuTeQ 0.8 alpha 7 software. This method, positioned between qualitative and quantitative analysis, enables the quantitative study of word frequency and co-occurrence, followed by qualitative interpretation of emerging word clusters [21].

The software lemmatised words, i.e., verbs were reduced to the infinitive, nouns to the singular, and adjectives to the masculine singular without disambiguation [22]. Analyses focused on active forms (adjectives, adverbs, common nouns, and verbs) as well as supplementary grammatical forms (articles and pronouns).

Subsequently, we conducted an analysis using Reinert’s method [23]. This analysis first divided the text into context units (CUs) according to the number of active forms occurrences and punctuation. Then, a simple hierarchical descendant classification (HDC) of the text was conducted, including a Factorial Correspondence Analysis (FCA) and χ² tests to assess the link to the three groups of participants. For the HDC, the number of terminal classes was set at 18 to detect more subtle differences, jointly determined by MR and LZ (Ph.D. candidate in health psychology, female) after several explorations. This decision reflects a methodological compromise between ensuring sufficient representativeness of the corpus and obtaining classes with adequately differentiated lexical content. With the default setting (10 classes), the analysis produced only three terminal classes, including one that accounted for nearly half of the corpus and exhibited low lexical differentiation. Increasing the number of requested classes improved the interpretability of the classification without generating overly fragmented classes. The HDC also indicates the variables associated with each class by indicating the *χ²*. We calculated Cramér’s V for each of these *χ²* values to evaluate the overall effect of the association on the entire sample, considering the classified text segments. These indicators were interpreted as described by Cohen [24]. Additionally, the FCA was performed on the HDC results to graphically visualise relationships between classes. The resulting factors were characterised by MR and LZ.

For each lexical class, co-occurrence similarity analyses were then performed (based on edge.betweeness.community community detection algorithms), and subclasses were created by grouping related communities, creating network analyses on associations between words in each class. These analyses create a graphical representation of lexical form co-occurrences, calculated via the intersection links between text segments, resulting in trees that identify central and peripheral words. The analytical process followed the recommendations of Montalescot et al. [25].

For international publication, the French terms were translated into English, preserving the semantic equivalence of the original synonyms. All translations were performed using the Cambridge Dictionary (https://dictionary.cambridge.org/fr/dictionnaire/anglais-francais/). The French version of the HDC table is provided in S1 Fig in S1 File.

Finally, qualitative inductive analyses according to the quantitative outputs were conducted independently by MR and LZ and merged. Qualitative analysis involved interpreting the quantitative output (*χ²* and figures): descriptions of the active, supplementary forms and associated verbatim of each class and subclass, inferences about their meaning, examination of the links between central and peripheral words in the similarity graphs, and explanation of the links to the associated variables. The two independent analyses were combined by reviewing each category and comparing interpretations. Any difficulties or disagreements were noted for inclusion in the results; a discussion session with other researchers familiar with the interviews (AN and KL) was planned in the event of persistent disagreements, should the need arise, but this proved unnecessary. These inductive analyses incorporated the researchers’ situated perspectives, and both MR and LZ wrote reflexive statements summarised as “intellectual equations” in Table 1 [25–27].

**Table 1:** Researchers’ intellectual equations.

Intellectual equations	LZ	MR
**Training and career background**	Ph.D. candidate in health psychology, focusing on lung cancer care and the implementation of cognitive behavioural therapy applications for insomnia in oncology. Holds a master’s degree in health psychology with an integrative approach.	Holds a master’s degree in public health, specialising in the epidemiology of communicable and non-communicable diseases in tropical regions. Research assistant studying health behaviours related to healthcare product use (vaccines, adjuvant treatments). Has contributed to projects on brain and ear, nose, and throat cancers.
**Lexical analysis experience and methodological reflexivity**	Experienced in qualitative (reflexive thematic) analysis; previously conducted Reinert analyses in parallel with her Ph.D. work. Emphasises a non‑post‑positivist stance. Also trained in quantitative methods.	First experience with textual statistical analysis (Reinert, similarity). Trained in quantitative and inductive thematic approaches; values mixed-method research integrating quantitative and qualitative data.
**Link to the ICEbreaker project**	Read the full corpus for this study but did not perform additional analyses.	Worked on qualitative data collected from relatives within the research project, conducted the inductive thematic analysis with AI, and compiled and prepared the full corpus for analysis.

Regarding contextual reflexivity, it is important to situate this research within the broader post-COVID context in France. The pandemic significantly accelerated the use of digital health applications among both HCPs and the population, including older adults. This widespread digital adoption reflects a broader European trend [28].

## Results

### Participants

Thirty-eight patients (including two with unusable interviews that could not be transcribed), 20 relatives, and 43 HCPs were interviewed. The average age of the participants was between 40 and 50 years, and the vast majority were women (n = 76; 76.77%) with a relatively high socioeconomic status. Most patients (participants or relatives) had breast cancer or relatives with breast cancer (n = 25; 44,64%). The relatives and patients both demonstrated good to very good digital perceived competencies. The HCPs had substantial experience working in oncology (n = 27; 62.79% with more than 10 years of experience), mostly full-time (n = 23; 53.49%). Their sociodemographic, medical, and digital characteristics are presented in Tables 2 and 3.

**Table 2:** Patients’ and relatives’ sociodemographic and clinical characteristics.

Characteristics	Patients(n = 36)	Relatives(n = 20)
**Age (years), m ± sd [min–max]**	49.86 ± 10.93[26–74]	45.5 ± 13.85[22–78]
**Gender, n (%)**		
Male	5 (13.89%)	3 (15.00%)
Female	31 (86.11%)	17 (85.00%)
**Education, n (%)**		
Diploma below high school level	2 (5.56%)	1 (5.00%)
High school diploma	3 (8.33%)	2 (10.00%)
Above high school level	31 (86.11%)	17 (85.00%)
**Socio-Professional Category, n (%)**		
Executives or intellectual professions	20 (55.56%)	11 (55.00%)
Intermediate occupations	4 (11.11%)	4 (20.00%)
Tradespersons, shop, or business owners	5 (13.89%)	1 (5.00%)
Farmers	–	–
Employees	5 (13.89%)	3 (15.00%)
Workers	–	–
Unemployed	–	0(0%)
Student	–	1(5.00%)
Other professional activity	–	0(0%)
**Perceived precarity** ^*^ **, n (%)**		
1	11 (30.56%)	6 (30.00%)
2	12 (33.33%)	6 (30.00%)
3	4 (11.11%)	7 (35.00%)
4	5 (13.89%)	1 (5.00%)
5	4 (11.11%)	0 (0.00%)
**Cancer (patients or relatives)** ^**^ **, n (%)**		
Abdomen	1 (2.78%)	–
Anus	1 (2.78%)	–
Breast	17 (47.22%)	8 (40.00%)
Cervix	1 (2.78%)	–
Colon	1 (2.78%)	–
Digestive	–	4 (20.00%)
Leukaemia	1 (2.78%)	–
Lymphoma	2 (5.56%)	–
Lung	2 (5.56%)	4 (20.00%)
Pancreas	1 (2.78%)	–
Brain	2 (5.56%)	1 (5.00%)
Haematologic	–	2 (10.00%)
Rectum	2 (5.56%)	–
Skin	1 (2.78%)	–
Urogenital	1 (2.78%)	3 (15.00%)
Inferior vena cava	2 (5.56%)	–
Cancer of unknown primary origin	1 (2.78%)	1 (5.00%)
**Health status (patients or relatives)** ^***^ **, n (%)**		
In treatment	14 (38.89%)	11 (50.00%)
In remission	22 (61.11%)	5 (22.73%)
Deceased	N/A	6 (27.27%)
**Relatives’ relationship, n (%)**		
Spouse	N/A	9 (42.86%)
Daughter	N/A	5 (23.81%)
Father	N/A	3 (14.29%)
Granddaughter	N/A	2 (9.52%)
Mother	N/A	1 (4.76%)
Sister	N/A	1 (4.76%)
**Treatment, n (%)**		
Chemotherapy	26 (72.22%)	20 (36.36%)
Radiotherapy	22 (61.11%)	13 (23.64%)
Surgery	28 (77.78%)	11 (20.00%)
Hormone therapy	12 (33.33%)	6 (10.91%)
Immunotherapy	3 (8.33%)	3 (5.46%)
Clinical trial	–	2 (3.64%)
**Perceived digital competencies, (%)**		
Very competent	79.38%	85.56%
Fairly competent	16.25%	10.00%
Neither	0.63%	3.33%
Not very competent	3.44%	0.56%
Not at all competent	0.31%	0.56%

*Participants rated their ability to “make ends meet” at the end of the month on a 5-point Likert scale, ranging from 1 (very easily) to 5 (very difficult); **Percentages may exceed 100% because some participants reported more than one type of cancer; ***Although 20 participants were surveyed, one participant reported three survivors (i.e. n = 22 survivors).

**Table 3:** HCPs sociodemographic characteristics.

Characteristics	HCPs (n = 43)
**Age (years), m ± sd [min-max]**	43.93 ± 10.19 [27-65]
**Gender, n (%)**	
Male	15 (34.88%)
Female	28 (65.12%)
**Education, n (%)**	
Diploma below high school level	
High school diploma	
Above high school level	43 (100%)
**Profession, n (%)**	
Physician	18 (41.86%)
Psychologist	9 (20.93%)
Nurse	6 (13.95%)
Health executive	4 (9.30%)
Social worker	2 (4.65%)
Physiotherapist	1 (2.33%)
Pharmacist	1 (2.33%)
Researcher-clinician	1 (2.33%)
Dietitian	1 (2.33%)
**Percentage of oncology work per week, n (%)**	
5–25%	3 (6.98%)
30–50%	6 (13.95%)
60–80%	11 (25.58%)
90–100%	23 (53.49%)
**Years working with patients with cancer, m ± sd, n (%)**	14.10 ± 8.21
0–5 years	4 (9.30%)
6–10 years	12 (27.91%)
> 10 years	27 (62.79%)
**Place of work, n (%)**	
Public hospital	31 (72.09%)
Private hospital	7 (16.28%)
Clinical practice	5 (11.63%)

Most interviews were conducted via videoconference and lasted an average of 43 minutes for patients, 46 minutes for relatives, and 37 minutes for HCPs.

### Textual statistical analysis

Four classes emerged from the analysis (Fig 1 and Table 4): (1) cancer experience, (2) patient care, (3) imagination and positioning in the conceptualisation of the digital tool: the content, and (4) tool shape: the container. Overall, MR and LZ agreed on the interpretation of the different classes. Few difficulties were identified, and these are specified below.

**Table 4:** Variables associated with classes of the hierarchical descending classification of the discourse analysis (Reinert’s analysis). ***p < 0.0001; **p < 0.01.

Classe 1		Classe 2		Classe 3		Classe 4	
*Variables*	*χ²*	*Variables*	*χ²*	*Variables*	*χ²*	*Variables*	*χ²*
Patients	173.56***	Relatives	352.36***	HCPs	20.06***	Patients	14.28***
Cramér’s V	0.16	Cramér’s V	0.22	Cramér’s V	0.05	Cramér’s V	0.04
Relatives	36.10***	Patients	−102.37***	Relatives	7.97**	HCPs	−9.84**
Cramér’s V	0.07	Cramér’s V	0.12	Cramér’s V	0.03	Cramér’s V	0.04
HCPs	−342.72***	HCPs	−103.09***	Patients	−39.86***		
Cramér’s V	0.22	Cramér’s V	0.12	Cramér’s V	0.07		

**Fig 1:**
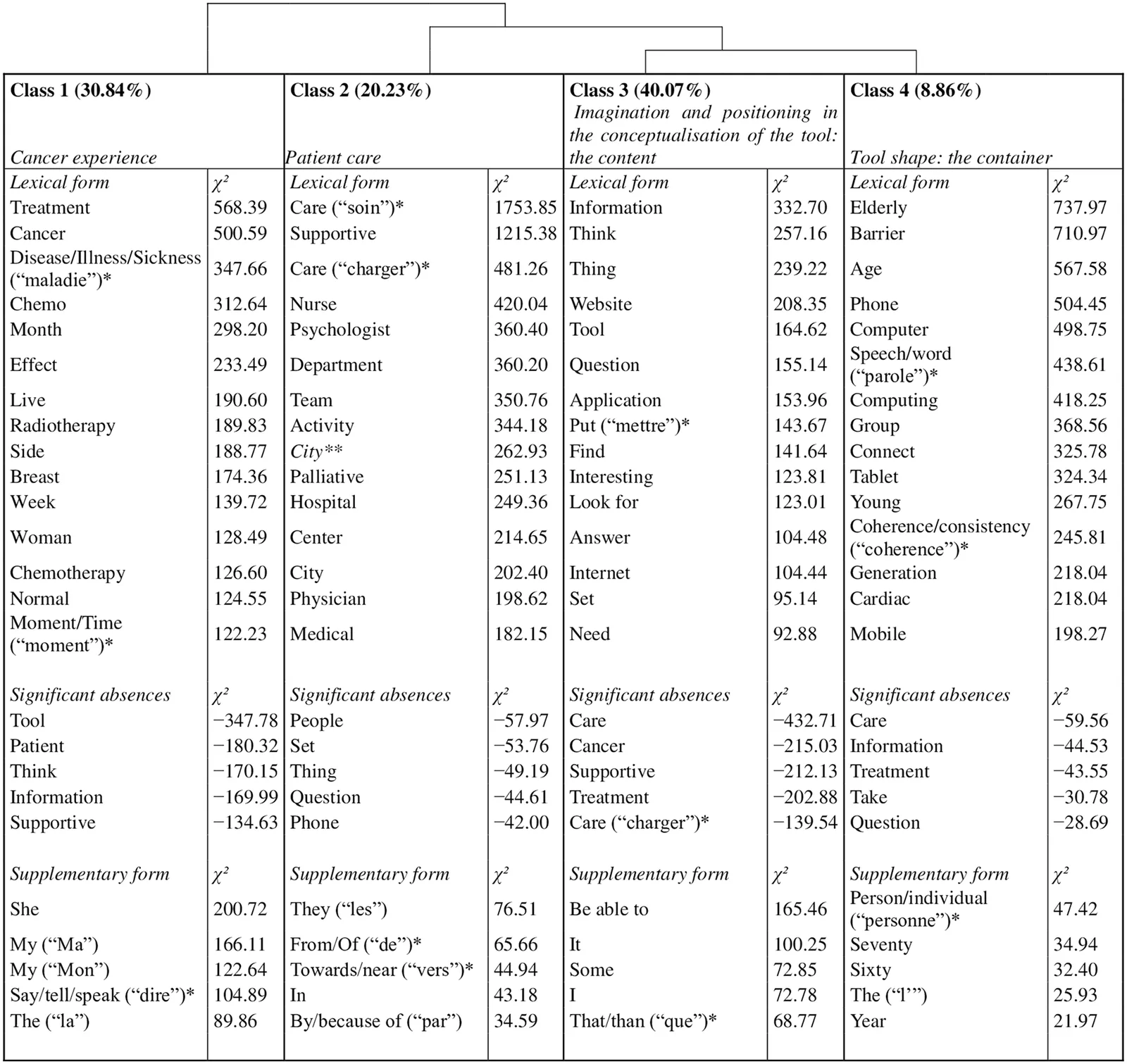
Representation of the hierarchical descending classification of the discourse analysis (Reinert’s analysis). *In French, these words can have two or more meanings; the translation is indicated in parentheses. **Words grouping together all the cities mentioned by patients (replaced in the transcripts); All *χ²* values are significant at *p* < 0.001.

### Class 1: Cancer experience

Class 1 encompasses 30.84% of the classified corpus and was the most specific class, representing the first to separate during the HDC. This class focuses on the lived experiences relating to the disease and its treatments, their duration, and effects. In the participants discourses, the need to move towards curative treatments was expressed, such as “radiotherapy” (*χ²  =* 189.83) or “chemotherapy” (*χ² =* 126.60). This class highlights the experience (“live”, *χ²  =* 190.60) of illness. The words “treatment” (*χ² =* 568.39) and “cancer” (*χ² =* 500.59) were the two most representative words in this class, which also form the basis of the two subclasses. The supplementary forms were relatively descriptive (“my”, “the”, “she”), demonstrating that this class is mainly descriptive but characterised by a prominent place of “emotions” (*χ²*  *=* 10.68, *p =* .001). The aim of the research, the creation of “tools” (*χ² =* −347.78, *p* < 0.0001) to improve “supportive” (*χ² =* −134.63, *p <* 0.0001) care, was not addressed at all in this class.

The verb “go” (*χ² =* 26.21*, p <* 0.0001) played a central role in this class (Fig 2), often used to refer to what the patient said or did, placing the patient at the centre of the action. As this term is central, we removed it from the similarity analysis to better highlight the different subclasses in the figure.

**Fig 2:**
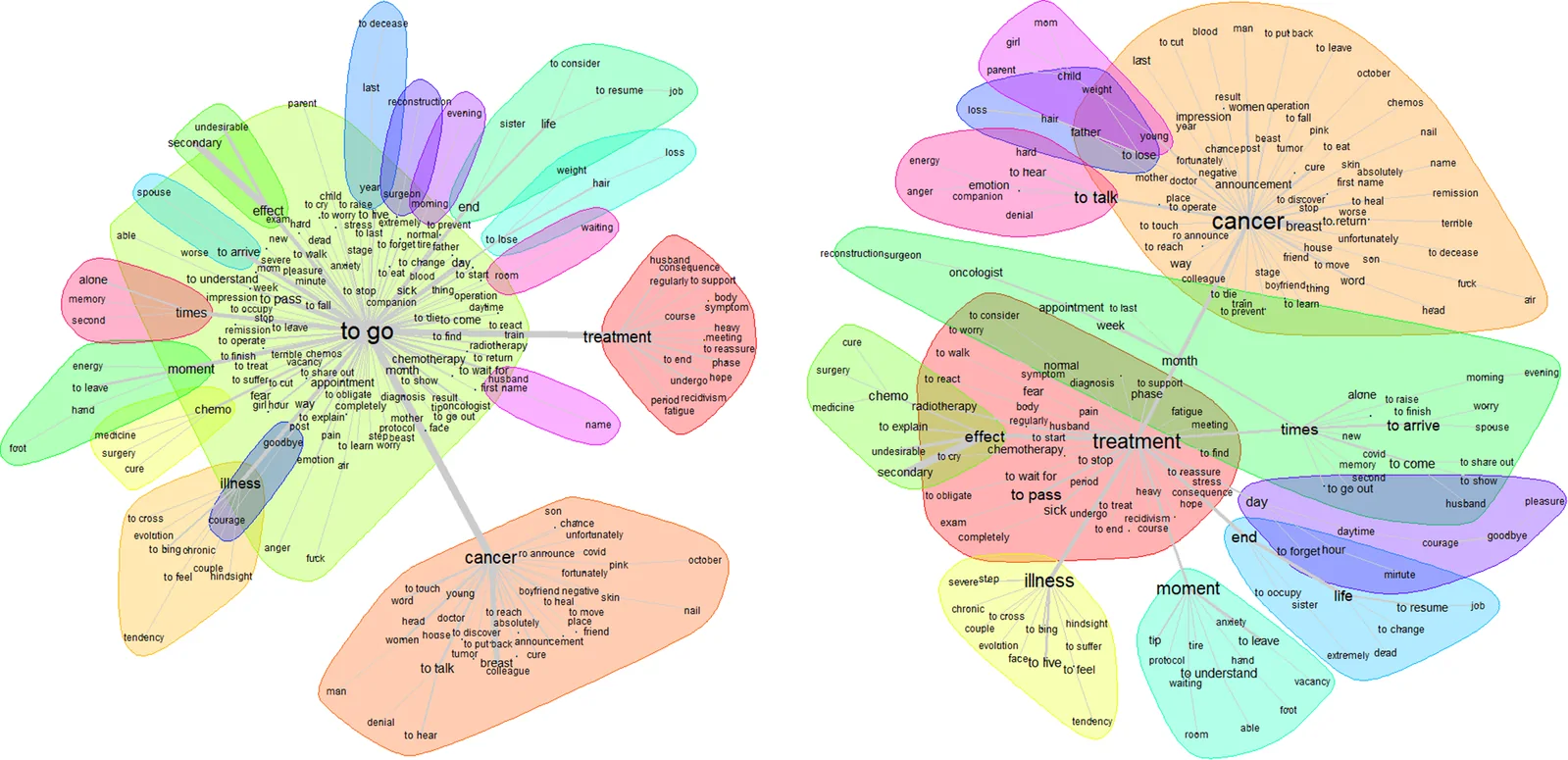
Similarity analysis of the HDC Class 1 results with and without “to go” (“aller”).

Class 1 was mentioned more often by patients (*χ² =* 173.56*, p <* 0.0001*, Cramér’s V  =* 0.16) and relatives (*χ² =* 36.10*, p <* 0.01*, Cramér’s V =* 0.07) and less often by HCPs (*χ² =*  − 342.72*, p <* .0001*, Cramér’s V =* 0.22). Addressing the experience of daily life with the disease appears, logically, to be more relevant to those receiving care than those providing it. It is also worth noting the significant absence of the word “patient” (*χ² =* −180.32*, p <* 0.0001), which clearly shows that this is not the discourse of HCPs. However, the effect of this variable remained weak (< 0.30), even negligible (< 0.10).

#### Subclass 1: The impacts and questions around cancer.

“Cancer” (*χ² =* 500.59) was at the heart of this first sub-theme. Participants generally reported difficulty talking (“talk”, *χ² =* 5.31*, p =* 0.021) about this subject with relatives (“friends” (*χ² =* 79.24*, p <* 0.0001; “partners” (*χ² =* 18.02*, p <* 0.0001) and the connection between these relatives, the disease, and the “emotions” associated with it (*χ² =* 10.68*, p =* 0.001). They expressed a fear of the direct consequences of the disease (“loss” (*χ² =* 14.25*, p =* 0.0002) of “hair” (*χ² =* 106.11*, p <* 0.0001) or “weight” (*χ² =* 12.79*, p =* 0.0003)), which was described as “terrible” (*χ² =* 19.83*, p <* 0.0001). Finally, the participants had many questions about the future, the risk of “dying” (*χ² =* 100.57*, p <* 0.0001), and the chances of “recovery” (*χ² =* 63.32*, p <* 0.0001).

“And so, for the first time, I thought to myself, maybe I won’t die right away from this cancer, and that there is a future ahead of me. This allowed me to approach the disease and treatments in a completely different way.” (Ella, 53 years old, relative who has had cancer)

#### Subclass 2: The treatment experience.

The second subclass is organised around “treatment” (*χ² =* 568.39). Here, too, participants reported “side” (*χ² =* 188.77*, p <* 0.0001) “effects” (*χ² =* 233.449, *p <* 0.0001), but referred more to the treatments themselves, such as “surgery” (*χ² =* 38.36, *p <* 0.0001) or “chemo” (*χ² =* 312.64). “Worries” (*χ² =* 52.64, *p <* 0.0001) and apprehension featured heavily, with questions about “suffering” (*χ² =* 34.06, *p <* 0.0001), “crying” (*χ² =* 56.58, *p <* 0.0001), and having a “chronic” (*χ² =* 35.07, *p <* 0.0001) illness. This subclass also evoked the temporality and timing of this illness: “appointments” (*χ² =* 2.34, *p =* 0.126) with “oncologists” (*χ² =* 54.84, *p <* 0.0001) and “surgeons” (*χ² =* 53.78, *p <* 0.0001), and the act of “coming” (*χ²  =* 18.83, *p  <* 0.0001) and “leaving” (*χ² =* 50.27*, p <* 0.0001).

“Needs change as the disease progresses, and they are not necessarily the same depending on the stage of treatment. I have a friend who has been undergoing intensive treatment, including chemotherapy, for almost two years.” (Pauline, 30 years old, patient talking about another patient)

“Anyway, after this video call, I’m exhausted! The fatigue is because I’ve finished my treatment since then, I haven’t had chemo since the end of May... I can only do one thing a day!” (Sandra, 53 years old, patient)

### Class 2: Patient care

Class 2 encompasses 20.23% of the classified corpus, focusing more on the definition of “supportive” (*χ² =* 1215.38*, p <* 0.0001) “care” (*χ² =* 1753.85*, p <* 0.0001), the two most representative words (Fig 3). Participants discussed the care provided by HCPs in general, notably nurses (*χ² =* 420.03*, p <* 0.0001) and psychologists (*χ² =* 360.40, *p <* 0.0001). The supplementary forms indicated that the discourse of this class positioned patients as individuals who go “towards/near” (*χ² =* 44.94, *p <* 0.0001) someone else to find care provided “by/because of” (*χ²* *=* 34.59, *p <* 0.0001) them. The participants did not talk about them directly, but rather generally referred to receiving care. In his analysis, MR highlighted that HCPs use the term “care” to refer to both supportive and palliative care.

**Fig 3:**
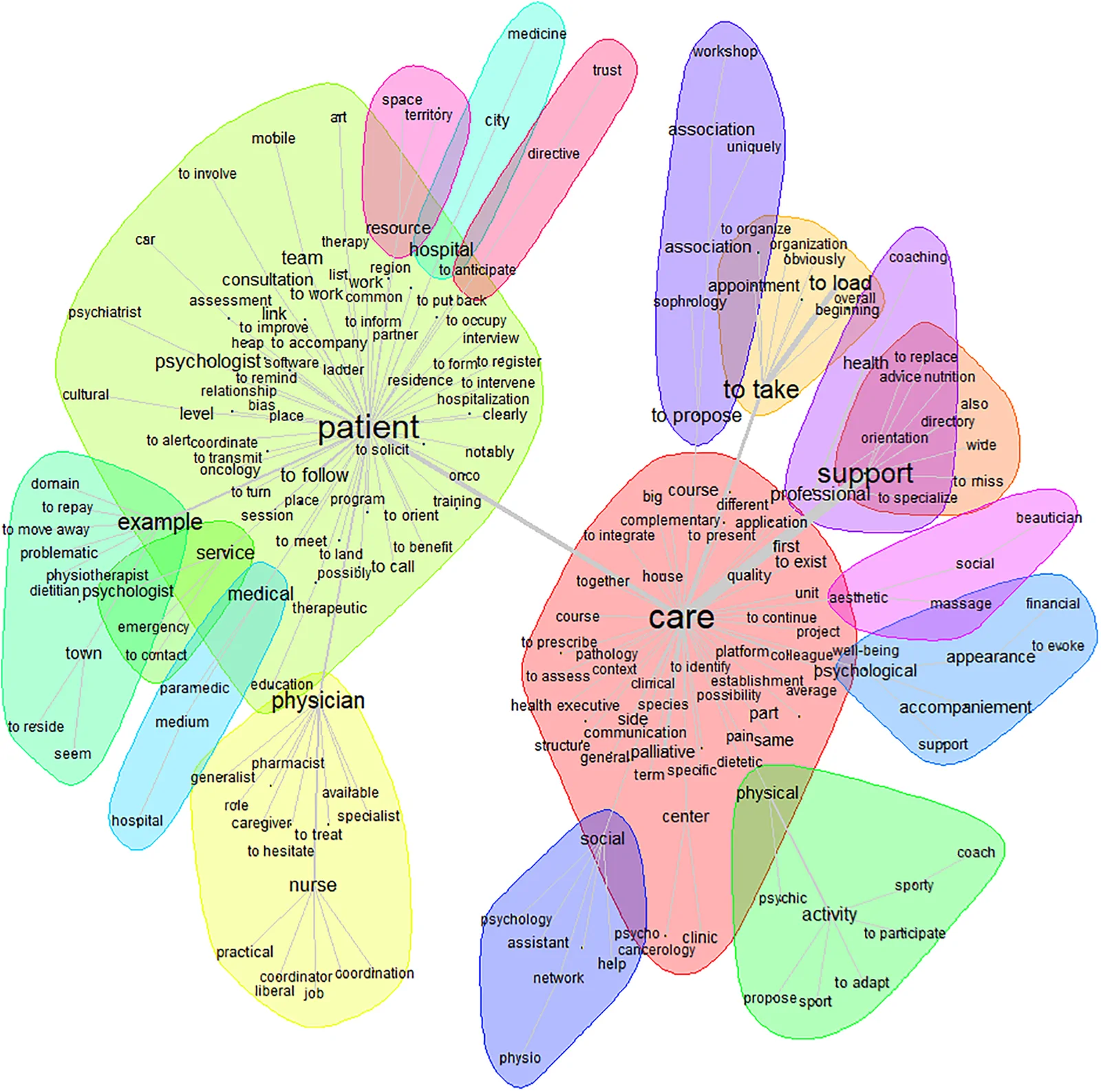
Similarity analysis of the HDC Class 2 results.

This class was mentioned more often by relatives (*χ² =* 352.36, *p <* 0.0001*, Cramér’s V  =* 0.22) and less often by patients (*χ² =* −102.37*, p <* 0.01*, Cramér’s V =* 0.12) and HCPs (*χ² =* −103.09*, p <* 0.0001*, Cramér’s V =* 0.12), indicating that supportive care was particularly emphasised in the relatives’ interviews. This was reflected in the supplementary forms used, which positioned the speaker as outside the action (« They » (*χ² =* 76.51*, p  <* 0.0001)), and in the absence of supplementary forms, such as “I” (*χ² =* −28.07*, p <* 0.0001). However, the effect of this variable remained weak (< 0.30).

#### Subclass 1: Supportive care provided to patients.

The first subclass focuses on the word “care” (*χ² =* 1753.85*, p <* 0.0001), particularly in the context of “supportive” (*χ² =* 1215.38*, p <* 0.0001). Participants discussed the various types of support available, including “psychological” (*χ² =* 128.36*, p <* 0.0001), “financial” (*χ²  =* 16.30*, p <* 0.0001), “physical” (*χ² =* 151.77*, p <* 0.0001), and “activities” (*χ²  =* 344.18*, p  <* 0.0001). This was possible through “associations” (*χ² =* 112.91*, p <* 0.0001) and HCPs. Participants occasionally referred to the idea of a “platform” (*χ² =* 2.41, *p =* 0.12), although this was not significantly associated with this class. The participants referred to everything that existed in terms of support to then discuss what they would need in terms of a support platform.

“For us, it’s basically, then... either effectively, through our interventions, particularly as a mobile team, we have... um... a mission to provide supportive care to patients in the sense of consultation, pain management, discomfort, etc.” (Claudia, 50 years old, psychologist)

#### Subclass 2: Patients at the centre of care.

In this second subclass, the word “patient” (*χ² =* 126.56*, p <* 0.0001) is central. Participants described the importance of being at the centre of care. Relationships with “physicians” (*χ² =* 192.62, *p <* 0.0001) and “nurses” (*χ² =* 420.04, *p <* 0.0001) were mentioned in the context of “department” (*χ² =* 360.20, *p <* 0.0001) and “hospital” (χ² = 249.36, *p*  < 0.0001) or general practitioner (“*médecin de ville”* (*χ² =* 202.40, *p <* 0.0001)). Participants gave numerous examples of follow-up and relationships, indicating that this requires many “resources” (*χ² =* 67.95, *p <* 0.0001).

“Because, afterwards, we’ll be focusing on oncological follow-up, but right now, we’re moving beyond the scope of supportive care. Well, it’s about optimizing patient care and optimizing their quality of life, above all else” (Paul, 38 years old, oncologist).

### Class 3: Imagination and positioning in the conceptualisation of the digital tool: the content

Class 3 encompasses 40.07% of the classified corpus, a large part of the discourse, which seems logical as it answers the research question by describing what a digital “tool” (*χ²  =* 164.62, *p <* 0.0001) should do. The main word in this class was “information” (*χ²  =* 332.70, *p <* .0001; Fig 4), highlighting one of the fundamental needs reported by participants: access to information. The second word was “think” (*χ² =* 257.16, *p <* 0.0001), which participants used particularly in the phrase “I think” and which shows that they give their opinions on the tool, with “I” (*χ² =* 72.78, *p <* 0.0001) also emerging as associated. The other supplementary forms were along the same lines, emphasizing “being able to” (*χ² =* 165.46*, p <* 0.0001) do this or that: people conceptualise what is possible to do. The significant absences of “supportive” (*χ² =* −212.13*, p <* 0.0001), “care” (*χ² =* −432.71, *p <* 0.0001), and “treatment” (*χ² = −*202.88, *p <* 0.0001) shows that this class considers novelty in care. MR and LZ noted that this class was more difficult to interpret because it was very large.

**Fig 4:**
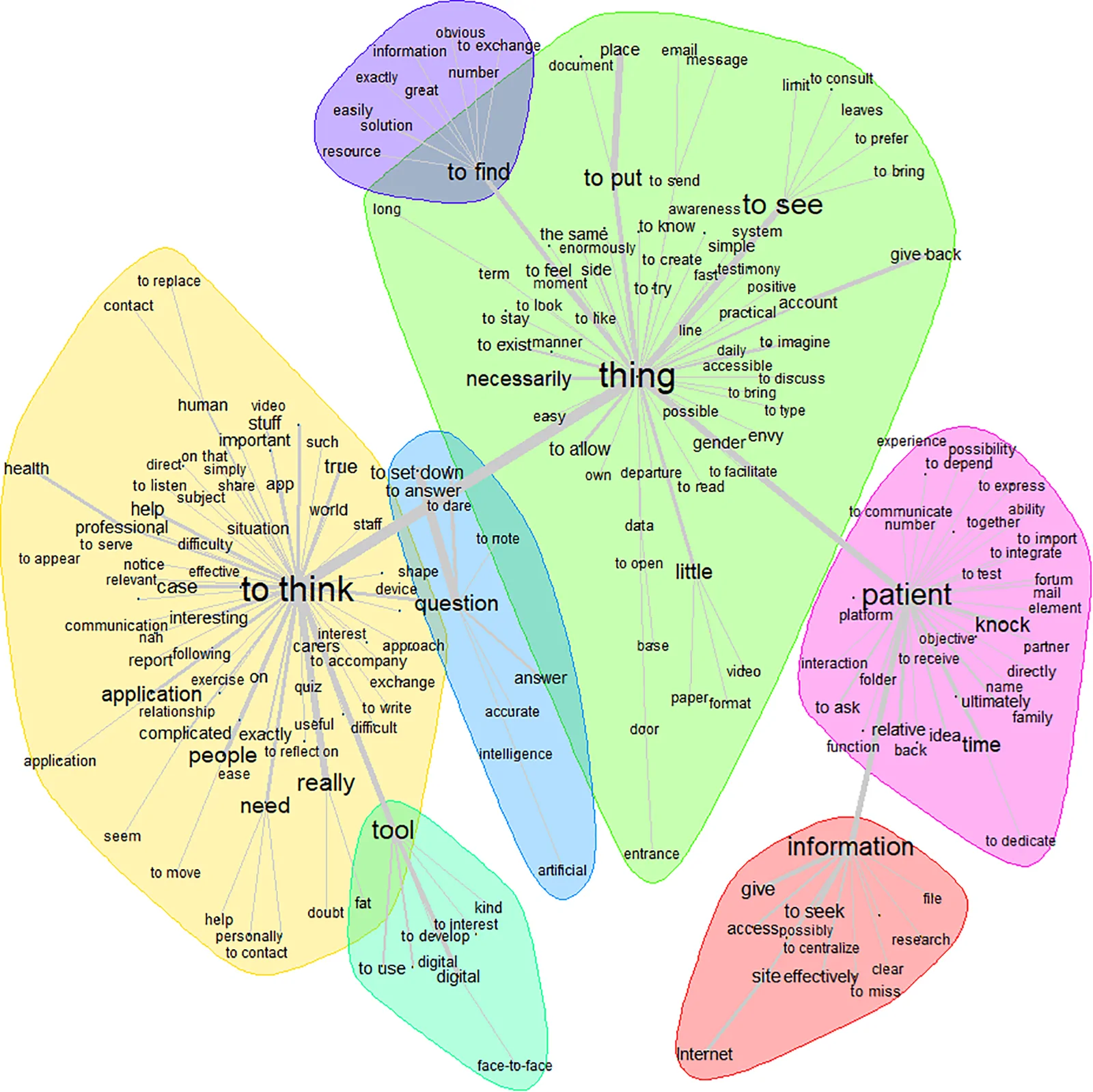
Similarity analysis of the HDC Class 3 results.

The effect of all variables associated with this class was negligible (< 0.10).

#### Subclass 1: Thinking about the tool.

This first subclass focuses on what participants “think” (*χ² =* 257.16*, p <* 0.0001) about the “digital” (*χ² =* 50.67, *p <* 0.0001) “tool” (*χ² =* 164.62, *p <* 0.0001). They emphasised a “need” (*χ² =* 92.88, *p <* 0.0001) for help and to be “personally” (*χ² =* 18.41, *p <* 0.0001) in “contact” (*χ²  =* 25.91, *p <* 0.0001). This last word highlights the importance of maintaining human contact and draws a parallel with “face-to-face” interactions (*χ² =* 5.97, *p =* 0.015). The participants also emphasised the need for information and answers to their “questions” (*χ²  =* 155.14, *p <* 0.0001) and “doubts” (*χ² =* 8.3*, p =* 0.004), but in a “precise” (*χ² =* 11.83*, p  <* 0.001) and “relevant” (*χ² =* 18.33, *p <* 0.0001) manner. The reliability of information was highlighted as important. The inclusion of “caregivers” (*χ² =* 54.06, *p <* 0.0001) in the application is highlighted to meet their needs, which are often neglected.

“So there’s a lack of information, a lack of support, a lack of many things in any case, from a human perspective, so it’s true that a digital tool can help, but there has to be someone behind it!” (Sophia, 55 years old, relative)

“I think so, because, in the end, um... there are also people who are more reluctant to trust information on the internet, or how can we be sure that this site is going to give us good information...” (Harper, 32 years old, relative)

#### Subclass 2: Elements to include in the tool.

The second subclass emphasises “things” (*χ² =* 239.22, *p <* 0.0001) to be included in the tool, such as “testimonials” (*χ² =* 19.14, *p <* 0.0001), “messages” (*χ² = 58.18, p <* 0.0001) with HCPs, and “questionnaires” (*χ² =* 25.92, *p <* 0.0001). Participants underlined that a tool must be “simple” (*χ² =* 45.01, *p <* 0.0001) to use, particularly in relation to everything that already “exists” (*χ² =* 33.74, *p <* 0.0001). The notion of “finding” (*χ² =* 141.64, *p <* 0.0001) things such as “info” (*χ² =* 28.58, *p <* 0.0001), telephone “numbers” (*χ² =* 22.83, *p <* 0.0001) or “solutions” (*χ² =* 24.01, *p <* 0.0001) was particularly emphasised.

“And so, um, I think that’s where information is needed to be able to help people, guide them, refer them to organisations that will be able to help them, that kind of thing, so...” (Gabriela, 51 years old, patient)

“I think digital technology can be good for bringing all the information together in one place with a system where you click on the social aspect, the legal aspect, the... and you get the information as it unfolds.” (Celia, 30 years old, patient)

#### Subclass 3: The tool must adapt to the user.

The third subclass, like subclass 2 of class 2, places the “patient” (*χ² =* 10.88, *p <* 0.001) at the centre of the discourse. It is the patient who must be given access to “information” (*χ²  =* 332.70*, p <* 0.0001), who must be able to “seek” (*χ² =* 123.01, *p <* 0.0001), “ask” (*χ²  =* 104.48, *p <* 0.0001), and “express” (*χ² =* 5.33, *p =* 0.02) themself. This gives “time” (*χ²  =* 31.74, *p <* 0.0001) to the patient, but also to HCPs, allowing them to adapt to the patient’s needs. This subclass also highlights the need for direct assistance to “relatives” (*χ² =* 16.25, *p  <* 0.0001).

“I think there are other things to work on before implementation, etc., but yeah, forums, why not, and what’s more, it would really be a dedicated platform for patients with cancer” (Chloe (HCP 23), 27 years old, social worker)

### Class 4: Tool shape: the container

Class 4 encompasses 8.86% of the classified corpus. It is a small class that remains closely associated with class 3, as these are the last two classes to be differentiated. It is organised around the themes of “barriers” (*χ² =* 710.97, *p <* 0.0001) to the use of a tool (*χ²  =* 162.39*, p <* 0.0001) and also how the tool should function and be accessible. Some words are significantly absent from this class; for example, there was no mention of “care” (*χ² =* −59.56, *p  <* 0.0001), “treatment” (*χ² = −*43.55, *p <* 0.0001) or “information” (*χ² =* −44.53, *p <* 0.0001). Thus, like class 3, this class substantially focuses on the topic of the research interview.

For the same reason, the word “tool” was central in the similarity analysis of this class (Fig 5); thus, as for the first class, we removed this word from the analysis to better visualise the different subclasses in the figure. Extracting subclasses from the similarity analyses was more complicated because of the complexity of the graph; however, MR and LZ both identified the same subclasses.

**Fig 5:**
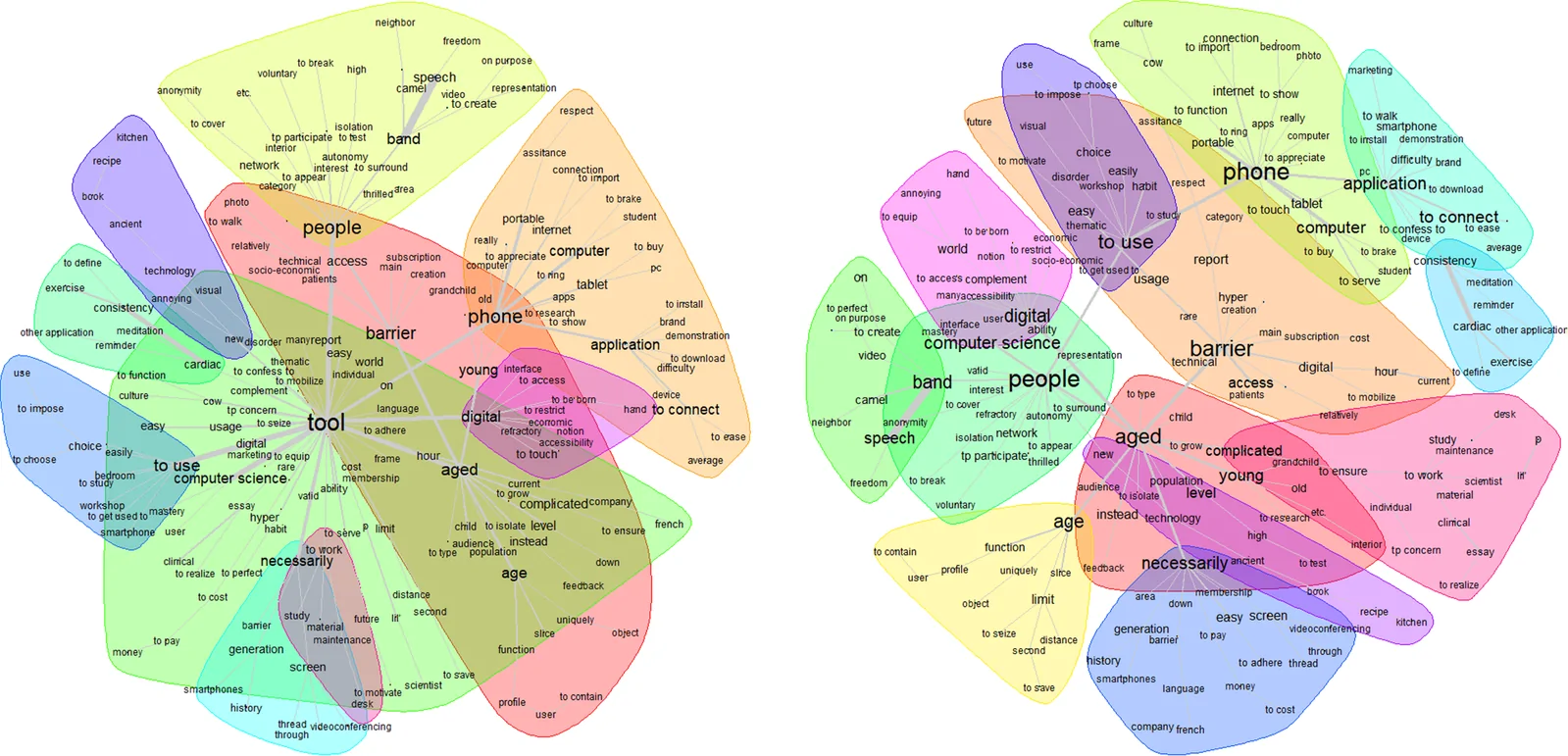
Similarity analysis of the DHC Class 4 results with and without “tool” (“outils”).

Similar to the findings for class 3, the effect of all variables associated with this class was negligible (< 0.10).

#### Subclass 1: The barriers faced.

The first subclass highlights the various “barriers” (*χ² =* 710.97, *p <* 0.0001) to using a tool, in particular “age” (*χ² =* 567.58, *p <* 0.0001) or being “elderly” (*χ² =* 737.97, *p <* 0.0001). Age also appeared in the supplementary forms, mainly numbers and the word “year” (*χ²  =* 21.97, *p <* 0.0001). These forms also demonstrated that participants mainly talk about others (“person/individual” (*χ² =* 47.42, *p <* 0.0001) or “the” (*χ² =* 25.93, *p <* 0.0001)), thus reporting more on the difficulties of others than their own. This is a barrier to accessing “digital” (*χ² =* 114.54, *p <* 0.0001) “technology” (*χ² =* 133.78*, p <* 0.0001). Participants reported that use can be complicated depending on “generations” (*χ² =* 218.04*, p <* 0.0001), access to the “language” (*χ² =* 40.46, *p <* 0.0001), “economic” (*χ² =* 32.61, *p <* 0.0001) level, and whether they enjoy using computers. The word “necessarily” (*χ² =* 4.19, *p =* 0.041) also stood out, but in a negative sense: participants qualified their statements, indicating that that age or profile is not necessarily an obstacle, as it depends greatly on the individual.

“Well, without necessarily having any preconceived notions, you know, for example, depending on age... Because I realise that there are some really elderly people who are very comfortable with online things” (Carolina, 42 years old, psychologist)

“But we know that there are simple cell phones for very elderly people that allow them to have emergency phones, their children’s numbers, things like that, without making a big deal out of it” (Sean, 74 years old, patient)

#### Subclass 2: The use of the tool itself.

For this second subclass, participants emphasised that the “application” (*χ² =* 9.27, *p*  < 0.01) should be used as a supplement and that patients and their relatives should be allowed to “choose” (*χ² =* 6.73, *p <* 0.01) whether to use it and what to do with it. Some may be “resistant” (*χ² =* 55.61, *p <* 0.0001) to using it. The importance of maintaining “anonymity” (*χ² =* 61.30, *p <* 0.0001) was also expressed.

Participants compared what could be offered with what already exists, and indicated the importance of “marketing” (*χ² =* 28.52, *p <* 0.0001) in implementing the application. They indicated that access must be possible via “phones” (*χ² =* 504.45, *p <* 0.0001) or “tablets” (*χ²  =* 324.34, *p <* 0.0001). Thus, many options could be offered, which ties in with class 3.

“And then we could add lots of things, we could add ideas to help patients with their diet, with our patients, with recipes, things like blended food!” (Brad, 50 years old, oncologist)

### Links between classes

Links between classes were analysed by FCA (Fig 6).

**Fig 6:**
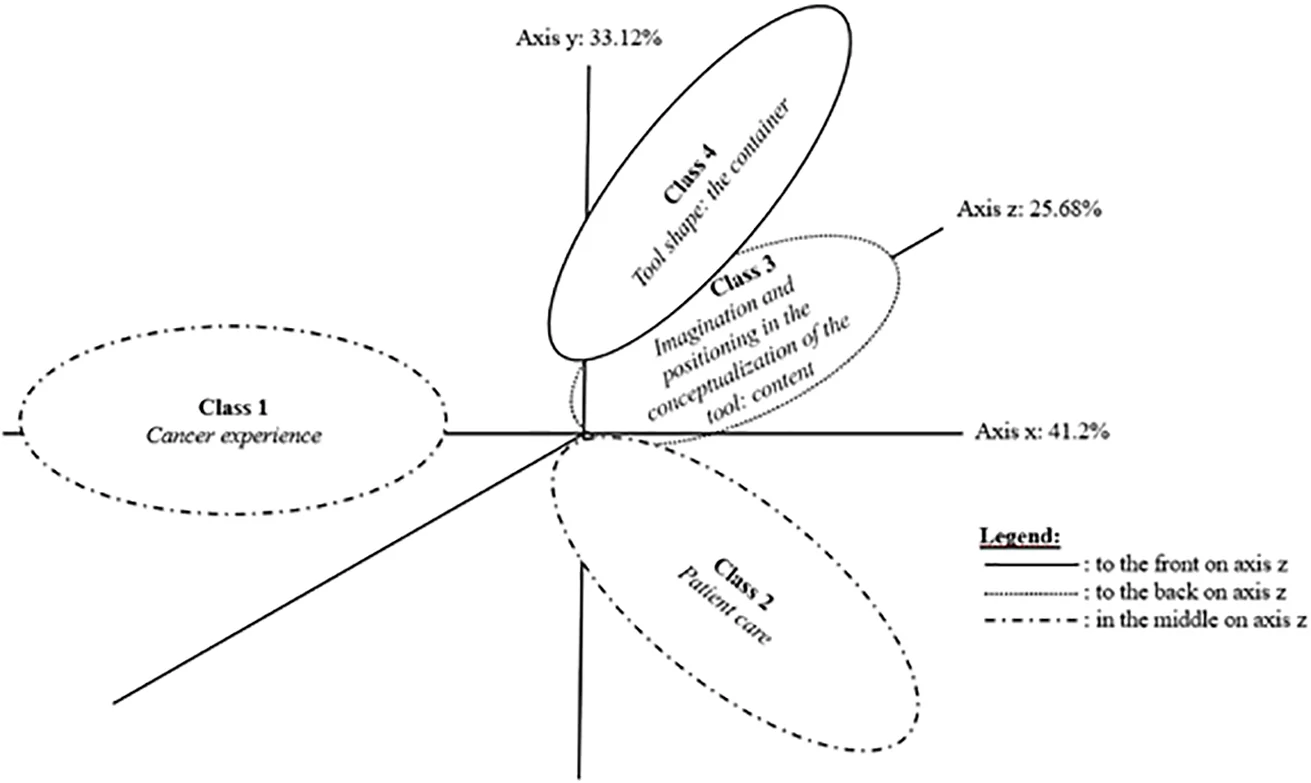
FCA of HDC results to identify links between classes.

Axis 1 (x) represents 41.2% of the differences between classes in the corpus. It contrasts class 1 with classes 2, 3, and 4. Thus, in the discourse, it opposes illness and treatment to the overall care of patients.

Axis 2 (y) represents 33.12% of the differences. It contrasts classes 3 and 4 with class 2. Class 1 is in the middle. Thus, the axis represents a continuum from what already exists (class 2) to what should or could exist with the new tool.

Axis 3 (z) represents 25.68% of the differences. It contrasts class 3 with class 4, leaving classes 1 and 2 in the middle. This last axis represents the contrast between the content and the container of the tool.

## Discussion

The present study highlights a fundamental shift in how digital supportive care tools are conceptualised by patients, relatives, and HCPs. Rather than being viewed as instruments of care or treatment, digital tools are primarily understood as informational, reflexive, and user‑centred spaces that support individuals in seeking information, articulating concerns, making choices, and preserving meaningful human support. This reconceptualisation underscores the importance of designing digital health interventions as customisable empowerment tools, inclusive of relatives, attentive to barriers of use, and intended to complement, rather than prescribe or replace existing care structures.

The textual statistical analysis identified four classes. The first two classes focused on everyday life with disease and supportive care, underscoring a perceived need for improvement in these domains, which were less frequently addressed by HCPs than by patients and relatives. Notably, no discourse focused on the experience of relatives themselves. Even when they contributed significantly to these classes (*e.g.,* Class 2), their accounts primarily centred on the patient’s care trajectory rather than on their own experiences. This absence should not be interpreted as a lack of needs or of willingness to express them, but rather as reflecting the limited space traditionally afforded to relatives’ voices within care and research contexts. This finding underscores the need to actively recognise relatives’ perspectives and develop dedicated forms of support for them. In contrast, Classes 3 and 4, differentiated at a later analytical stage and, thus, closely related, the focus shifted away from care and treatment toward the conceptualisation of the digital tool itself. Notably, the participants did not frame the tool as a clinical or therapeutic device, but as an informational, reflexive, and adaptive space enabling users to seek information, express concerns, make choices, and maintain human mediation. Class 4 further highlighted barriers to use and implementation, particularly for older individuals or those less comfortable with digital technologies. However, the literature shows that older people can use digital tools effectively if they receive adequate support [29]. Across patients, relatives, and HCPs, there was strong convergence regarding both identified needs and proposed solutions, pointing to the emergence of a digital tool conceived as an optional, patient‑centred complement to existing care structures, rather than a substitute. Although participants acknowledged potential barriers for others, such as limited access or digital literacy, they reported few barriers to their own use.

These findings are consistent with thematic analyses conducted on each of the three groups [18–20]. The textual statistical analysis demonstrated that HCPs tend to focus more on the tool itself than on the lived experience of those with the disease, which directly aligns with the findings of the previous HCP-focused study [19,20], in which the themes clearly emphasised the tool and its implementation. Maintaining a link with HCPs through the application appears to be a key expectation. The tool is expected to be highly personalised, along with providing clear and concise information. Consistent with the thematic analysis of patient interviews [18], the notion of temporality did not emerge as a prominent theme. Instead, digital tools were perceived as useful across all stages of the disease, consistent with the need to address the loss of connection experienced at the end of care reported in the literature for various cancer patient populations [30–32]. Furthermore, the effective use of such tools requires adequate eHealth literacy, defined as “the ability to seek, find, understand, and appraise health information from electronic sources and apply the knowledge gained to addressing or solving a health problem” [33].

Taken together, these findings address the study’s second aim by identifying key barriers (limited digital literacy linked to the age, concerns about data protection) and facilitators, including ease of access, personalisation, and the preservation of human mediation through HCP follow-up.

Within the Unified Theory of Acceptance and Use of Technology (UTAUT) model [34], particularly the extension of this model by Schretzlmaier et al. [35], the use of digital tools is influenced by habits and facilitating conditions. This perspective is reflected in participants’ comments, which emphasise the importance of easy access to information and an appropriate, user-friendly interface. These acceptance factors highlighted in UTAUT can be situated within a broader implementation framework, in particular through the Consolidated Framework for Implementation Research (CFIR; [36]), which highlights the need to leverage characteristics of the intervention itself, such as avoiding complexity, to support implementation. Beyond intervention characteristics, the CFIR identifies four domains that may influence implementation, including external factors (*e.g.,* patient needs and available resources, which were explored in this study and align with UTAUT’s facilitating conditions), the characteristics of the inner setting (notably, organisational norms and values, including support from patients and relatives partners, a point to which we return below), individual characteristics (which map onto UTAUT’s habits and acceptance factors), and the implementation process itself. Although not all of these domains were addressed here, combining UTAUT with CFIR provides a useful lens for understanding how design, context, and process interact in shaping the uptake of digital health tools.

The importance attributed to maintaining a relationship, even when mediated by digital tools, echoes the concept of digital alliance developed by Stern et al. [37]. Although a therapeutic relationship with a technological object cannot be assumed, an alliance may be established if the tool is perceived as credible and appropriate. Participants expressed expectations for human follow-up and interaction. These findings are also consistent with safety criteria for electronic patient-reported outcomes [38]. Participants highlighted data protection and security as essential conditions for a tool to be considered safe.

### Strengths and limitations

A major strength of this study lies in its examination of the discourse of patients, relatives, and HCPs regarding the development of a digital tool. This approach provides insight into shared expectations as well as differences between groups. The analysis covered more than 90% of the discourse, which is a particularly high classification rate compared with that of previous studies using similar methods, where classified units ranged from 63% to 87.74% [21,39–42].

This study has various limitations. First, the participants were of relatively high socio-economic status across all groups, as self-reported via their profession, education, and perceived precarity. In the adult age group, and the patients and their relatives demonstrated a high level of computer skills. Future studies should investigate these aspects in more precarious, older people with lower levels of education, given that these individuals generally have less access to cancer care [43]. Second, some patients had previously been relatives of individuals with cancer, while some relatives had themselves experienced cancer. This overlap of roles may have blurred the distinction between patient and caregiver perspectives in the discourse. Finally, from a methodological standpoint, the analysis was conducted in French, and the translation into English for publication can cause certain nuances to be lost. To counter this, we have tried to make our translations as clear as possible and have included the French language analyses in the supplementary Fig.

### Practice and research implications

The findings of this study provide a better understanding of the expectations of the three groups surveyed regarding the implementation of digital tools. It remains particularly important to co-develop these tools with patients, their relatives, and HCPs. In addition, the implementation of tools could be accompanied by therapeutic education to improve patients’ and relatives’ eHealth literacy, thus facilitating adoption [33]. Furthermore, the reported importance of human connection through the application raises questions about the interface to be used. One possible option would be to designate a person specifically responsible for responding to users and providing support when the tool reaches its limits. Although this approach would directly address the needs expressed by participants, it would likely be costly and difficult to implement within most healthcare settings. An alternative could involve the use of specialised artificial intelligence (AI), which has seen increasing adoption in recent years. However, the use of AI raises numerous ethical issues related to transparency, maintaining a human touch, and data security [44], along with environmental concerns. These considerations lead to a critical question: does the potential benefit of using AI outweigh its possible negative effects? Beyond AI, a complementary approach could involve engaging patients and relatives as partners in the implementation of the tool. The benefits of such involvement are well‑documented, with patient and relative engagement shown to enhance the quality and foster innovation in healthcare interventions [45]. With their lived experience of the disease and care, these individuals can contribute unique insights and support implementation by drawing on their experiential knowledge, understood as the knowledge derived from living with illness daily, navigating the care journey, relating to healthcare systems, and coping with the vulnerability that accompanies it [46], as they already do in the implementation of research initiatives [47]. To fulfil this role effectively, patient and relative partners should be recruited for clearly defined positions. Such professionalisation must remain attentive to existing power dynamics within healthcare, as well as to ongoing debates regarding the preservation of experiential knowledge and identity versus the risk of excessive normalisation through training or tokenism, understood here as the instrumentalisation of patients as mere symbolic partners rather than genuine collaborators [48]. At the same time, genuine recognition (including appropriate remuneration, clear role definitions, and adequate training) is central. Training should also equip partners to protect themselves, establish boundaries, and resist performance- and profitability-driven demands that are incompatible with patient partnership and healthcare values. This perspective aligns with the need to view patients and their relatives not only as being at the centre of care, like in our results, but also as active participants within the care process itself, in line with the Montreal Model of patient engagement [49–52].

Translating this vision into practice, one key recommendation emerging from this study is to move beyond *ad hoc* involvement and toward the systematic integration of patient partnership throughout the development, implementation, and evaluation of digital health tools. Rather than being limited to consultation or end-user testing, patients (and where relevant, relatives) should be fully engaged as members of the development team and as co-researchers, contributing to decision-making processes, priority setting, and the interpretation of use-related challenges, in accordance with the principles of justice and equity that underpin meaningful partnership [53]. Their competences and experiential knowledge are particularly critical for ensuring that digital tools remain aligned with real-life needs, preserve meaningful human mediation, and avoid reinforcing overly technocentric or prescriptive models of care. Embedding patients or relatives partners within clinical and research teams may also facilitate adoption in practice by fostering trust, relevance, and acceptability among future users. In this sense, this collaborative approach should not be viewed as an optional or ethical add-on, but as a core clinical strategy for designing digital tools that genuinely support empowerment, adaptability, and continuity of care [54].

From a research perspective, further investigation is needed to better capture the experiences and expectations of populations that are often underrepresented in digital health research, particularly older adults with limited computer skills and individuals with lower socioeconomic status. Exploring their specific perspectives would enable the more precise identification of usability requirements, access constraints, and support needs, thereby helping to prevent the reinforcement of existing health and digital inequalities. Beyond issues of access, there remains a significant gap in the literature regarding why and how patients choose to engage with digital health tools, including the factors that shape initial uptake, sustained use, or disengagement over time. Thus, future research should move beyond evaluations of effectiveness alone and examine the social, contextual, and relational determinants of digital tool adoption. In this regard, in Germany, access to validated digital tools through prescription and reimbursement mechanisms exists through the German Digital Health Applications (DiGA) system. The study of structured implementation frameworks such as DiGA could provide valuable insights into how institutional endorsement, clinical integration, and financial coverage influence patients’ trust, use patterns, and long-term engagement with digital health interventions [55].

## Conclusions

This study contributes to a more nuanced understanding of the needs surrounding digital tools designed to support patients and their relatives, indicating that such tools should be user‑centred, accessible, and tailored to individual needs. The textual statistical analysis across patient, relative, and HCP discourses demonstrates a strong convergence of expectations regarding the informational, supportive, and relational functions such a tool should fulfil. At the same time, this research draws attention to the risk of digital exclusion, particularly for older individuals and those with lower levels of digital literacy or socioeconomic resources. Addressing these inequalities requires not only thoughtful design but also adequate support, education, and integration within care systems.

Future developments and research should prioritise co-design approaches, ensuring that digital innovations genuinely enhance care experiences rather than inadvertently widening existing disparities.

## Supporting information

S1 File Supporting Information Captions Supplementary figures 1 to 6. Software releases in French. (DOCX)

## References

[1] Bansard E, Bouleuc C, Gaille M, Dolbeault S. Définir les soins de support : Une contribution philosophique. Psycho-Oncol. 2021;15(3):85–92. doi: 10.3166/pson-2021-0165

[2] Al Maqbali M, Al Sinani M, Al Naamani Z, Al Badi K, Tanash MI. Prevalence of fatigue in patients with cancer: A systematic review and meta-analysis. J Pain Symptom Manage. 2021;61(1):167-189.e14. doi: 10.1016/j.jpainsymman.2020.07.037 32768552

[3] Cerulla Torrente N, Navarro Pastor J-B, de la Osa Chaparro N. Systematic review of cognitive sequelae of non-central nervous system cancer and cancer therapy. J Cancer Surviv. 2020;14(4):464–82. doi: 10.1007/s11764-020-00870-2 32146576

[4] Rovito MJ, Chauhan KM, Baig H. Chemobrain and cancer survivorship: a scoping review of the literature. Cancer Medicine. 2025;14:e71383. doi: 10.1002/cam4.71383 PMC12611312 41223143

[5] Carrera A, Lettieri E, Lietti G, Martignoni S, Sgarbossa C, Cafazzo J. Therapies go digital. What drives physicians’ acceptance?. PLoS ONE. 2024;19. doi: 10.1371/journal.pone.0303302 PMC11086840 38728346

[6] Eggins R, Fowler H, Cameron J, Aitken JF, Youl P, Turrell G, et al. Supportive care needs and psychosocial outcomes of rural versus urban women with breast cancer. Psychooncology. 2022;31(11):1951–7. doi: 10.1002/pon.5977 35726399

[7] Fayet Y, Chasles V, Ducimetière F, Ray-Coquard PI. Le territoire, générateur d’inégalités face aux cancers. Revue francophone sur la santé et les territoires. 2018. doi: 10.4000/rfst.700

[8] Chemtob M-CC. Parcours de soins rénové, e-santé et maillage officinal: une réponse complexe à la pénurie de médecins. Médecine & Droit. 2024;2024: 122–8. doi: 10.1016/j.meddro.2024.07.003

[9] Kirkpatrick S, Davey Z, Wright PR, Henshall C. Supportive eHealth Technologies and Their Effects on Physical Functioning and Quality of Life for People With Lung Cancer: Systematic Review. J Med Internet Res. 2024;26:e53015. doi: 10.2196/53015 39059003 PMC11316152

[10] Fournier V, Duprez C, Grynberg D, Antoine P, Lamore K. Are digital health interventions valuable to support patients with cancer and caregivers? An umbrella review of web-based and app-based supportive care interventions. Cancer Med. 2023;12(23):21436–51. doi: 10.1002/cam4.6695 37937812 PMC10726780

[11] Zanni L, Journiac J, Savard J, Guerdoux E, Paillard-Brunet G, Fasse L, et al. Role and perception of healthcare professionals in referring cancer patients to online cognitive behavioral therapy for insomnia. Support Care Cancer. 2025;33(3):239. doi: 10.1007/s00520-025-09284-1 40021529

[12] Amat-Fernandez C, Garin O, Luer-Aguila R, Pardo Y, Briseño R, Lizano-Barrantes C, et al. Systematic review of the needs and health-related quality of life domains relevant to people surviving cancer in Europe. Qual Life Res. 2025;34(4):913–36. doi: 10.1007/s11136-024-03884-w 39847267 PMC11982114

[13] Hui D. Definition of supportive care: does the semantic matter?. Curr Opin Oncol. 2014;26(4):372–9. doi: 10.1097/CCO.0000000000000086 24825016 PMC12034444

[14] Paterson C, Robertson A, Smith A, Nabi G. Identifying the unmet supportive care needs of men living with and beyond prostate cancer: A systematic review. Eur J Oncol Nurs. 2015;19(4):405–18. doi: 10.1016/j.ejon.2014.12.007 25613370

[15] Zhu Y, Pei X, Chen X, Li T. Family Caregivers’ Experiences of Caring for Advanced Cancer Patients: A Qualitative Systematic Review and Meta-synthesis. Cancer Nurs. 2023;46(4):270–83. doi: 10.1097/NCC.0000000000001104 35482525

[16] Baudry A-S, Charton E, Piessen G, Vanlemmens L, Cortot A, Ceban T, et al. Emotional distress, supportive care needs and age in the prediction of quality of life of cancer patients’ caregivers: A cross-sectional study. Eur J Oncol Nurs. 2023;64:102324. doi: 10.1016/j.ejon.2023.102324 37146349

[17] Braun V, Clarke V. Using thematic analysis in psychology. Qualitative Res Psychol. 2006;3(2):77–101. doi: 10.1191/1478088706qp063oa

[18] Fournier V, Caton L, Laroussi-Libeault L, Aelbrecht-Meurisse C, Rybarczyk C, Richez L. A thematic analysis of semi-structured interviews exploring attitudes and needs for digital supportive care among patients with cancer and survivors. Sci Rep. 2026. doi: 10.1038/s41598-026-60210-9

[19] Naeyaert A, Fournier V, Laroussi-Libeault L, Haerinck J, Justin P, Duprez C, et al. Healthcare Professionals’ Perceptions of the Implementation of Psychosocial eHealth Tools in Oncology: A Qualitative Study. European Journal of Cancer Care. 2026;2026(1). doi: 10.1155/ecc/8841407

[20] Naeyaert A, Fournier V, Laroussi-Libeault L, Rebillon M, Dubart C, Justin P, et al. A qualitative study on relatives’ perceptions of digital supportive care tools in oncology. Eur J Oncol Nurs. 2026;84:103280. doi: 10.1016/j.ejon.2026.103280 42531798

[21] Montalescot L, Rascle N, Combe C, Untas A. Patients’ experience one year after dialysis initiation: a lexicometric analysis. Health Psychol Behav Med. 2021;9(1):380–97. doi: 10.1080/21642850.2021.1918552 34104566 PMC8158193

[22] Loubère L, Ratinaud P. Documentation IRaMuTeQ 0.6 alpha 3 version 0.1. 2014. http://www.iramuteq.org/documentation/fichiers/documentation_19_02_2014.pdf

[23] Reinert M. Une méthode de classification descendante hiérarchique : application à l’analyse lexicale par contexte. Cahiers de l’analyse des données. 1983;8:187–98.

[24] Cohen J. Statistical power analysis for the behavioral sciences. 2 ed. New York, NY: Psychology Press. 1988.

[25] Montalescot L, Lamore K, Cecile F, Untas A. What is the place of interpretation in text analysis? An example using ALCESTE® software. Qualitative Research in Psychology. 2024;:1–26. doi: 10.1080/14780887.2024.2316624

[26] Braun V, Clarke V. Thematic analysis: a practical guide. Los Angeles, London, New Delhi, Singapore, Washington DC, Melbourne: SAGE. 2022.

[27] Olmos-Vega FM, Stalmeijer RE, Varpio L, Kahlke R. A practical guide to reflexivity in qualitative research: AMEE Guide No. 149. Medical Teacher. 2023;45:241–51. doi: 10.1080/0142159X.2022.2057287 35389310

[28] Heponiemi T, Virtanen L, Kaihlanen A-M, Kainiemi Päivikki Koponen E, Koskinen S. Use and changes in the use of the Internet for obtaining services among older adults during the COVID-19 pandemic: A longitudinal population-based survey study. New Media & Society. 2022;26(6):3146–67. doi: 10.1177/14614448221097000

[29] Wilson J, Heinsch M, Betts D, Booth D, Kay-Lambkin F. Barriers and facilitators to the use of e-health by older adults: a scoping review. BMC Public Health. 2021;21(1):1556. doi: 10.1186/s12889-021-11623-w 34399716 PMC8369710

[30] Heveran L, Andrewes T. Nursing measures to support the needs of haematological cancer survivors post-treatment: a literature review. Br J Nurs. 2020;29(3):S18–24. doi: 10.12968/bjon.2020.29.3.S18 32053451

[31] Bamidele O, McGarvey H, Lagan BM, Ali N, Chinegwundoh Mbe F, Parahoo K, et al. Life after prostate cancer: A systematic literature review and thematic synthesis of the post-treatment experiences of Black African and Black Caribbean men. Eur J Cancer Care (Engl). 2018;27(1):10.1111/ecc.12784. doi: 10.1111/ecc.12784 29034575

[32] Laporte C, Vaure J, Bottet A, Eschalier B, Raineau C, Pezet D, et al. French women’s representations and experiences of the post-treatment management of breast cancer and their perception of the general practitioner’s role in follow-up care: A qualitative study. Health Expect. 2017;20(4):788–96. doi: 10.1111/hex.12518 27899006 PMC5513018

[33] Milanti A, Norman C, Chan DNS, So WKW, Skinner H. eHealth Literacy 3.0: Updating the Norman and Skinner 2006 Model. J Medical Internet Res. 2025;27:e70112. doi: 10.2196/70112 PMC11937704 40068166

[34] Venkatesh V, Morris MG, Davis GB, Davis FD. User Acceptance of Information Technology: Toward A Unified View1. MIS Quarterly. 2003;27(3):425–78. doi: 10.2307/30036540

[35] Schretzlmaier P, Hecker A, Ammenwerth E. Extension of the Unified Theory of Acceptance and Use of Technology 2 model for predicting mHealth acceptance using diabetes as an example: a cross-sectional validation study. BMJ Health Care Inform. 2022;29(1):e100640. doi: 10.1136/bmjhci-2022-100640 36379608 PMC9668013

[36] Kirk MA, Kelley C, Yankey N, Birken SA, Abadie B, Damschroder L. A systematic review of the use of the Consolidated Framework for Implementation Research. Implement Sci. 2016;11:72. doi: 10.1186/s13012-016-0437-z 27189233 PMC4869309

[37] Stern E, Breton Z, Alexaline M, Geoffroy PA, Bungener C. Redefining the relationship in digital care: A qualitative study of the Digital Therapeutic Alliance. Encephale. 2025;51(3):227–32. doi: 10.1016/j.encep.2024.02.011 38724431

[38] Denis F, Krakowski I. How Should Oncologists Choose an Electronic Patient-Reported Outcome System for Remote Monitoring of Patients With Cancer?. J Med Internet Res. 2021;23(9):e30549. doi: 10.2196/30549 34499046 PMC8461535

[39] Bienemann B, Barbosa AR, Cruz LVM da, Multedo M, Mograbi D. Health Benefits and Positive Acute Effects of Psilocybin Consumption: A Quantitative Textual Analysis of User Self-Reported Data. J Psychoactive Drugs. 2024;56(3):324–32. doi: 10.1080/02791072.2023.2226414 37348116

[40] Bucci F, Vanheule S. Families of adult people with disability: Their experience in the use of services run by social cooperatives in Italy. Int J Soc Welfare. 2017;27(2):157–67. doi: 10.1111/ijsw.12283

[41] Lamore K, Vioulac C, Fasse L, Flahault C, Quintard B, Untas A. Couples’ Experience of the Decision-Making Process in Breast Reconstruction After Breast Cancer: A Lexical Analysis of Their Discourse. Cancer Nurs. 2020;43(5):384–95. doi: 10.1097/NCC.0000000000000708 31033513

[42] Moscato A, Varescon I. Satisfaction and difficulties of French professional home caregivers in supporting older people with Alzheimer’s disease or alcohol misuse. Health Soc Care Community. 2018;26(1):27–34. doi: 10.1111/hsc.12457 28585296

[43] Jochum F, Hamy A-S, Gougis P, Dumas É, Grandal B, Laas E, et al. Effects of gender and socio-environmental factors on health-care access in oncology: a comprehensive, nationwide study in France. EClinicalMedicine. 2023;65:102298. doi: 10.1016/j.eclinm.2023.102298 37965434 PMC10641482

[44] Badawy W, Zinhom H, Shaban M. Navigating ethical considerations in the use of artificial intelligence for patient care: A systematic review. Int Nurs Rev. 2025;72(3):e13059. doi: 10.1111/inr.13059 39545614

[45] Baines R, Bradwell H, Edwards K, Stevens S, Prime S, Tredinnick-Rowe J, et al. Meaningful patient and public involvement in digital health innovation, implementation and evaluation: A systematic review. Health Expect. 2022;25(4):1232–45. doi: 10.1111/hex.13506 35526274 PMC9327849

[46] Gross O, Gagnayre R. Caractéristiques des savoirs des patients et liens avec leurs pouvoirs d’action: implication pour la formation médicale. Revue française de pédagogie Recherches en éducation. 2017; 71–82. doi: 10.4000/rfp.7266

[47] Bisson M, Aubrey-Bassler K, Chouinard M-C, Doucet S, Ramsden VR, Dumont-Samson O, et al. Patient engagement in health implementation research: A logic model. Health Expect. 2023;26(5):1854–62. doi: 10.1111/hex.13782 37309078 PMC10485341

[48] Montreuil M, Martineau JT, Racine E. Exploring Ethical Issues Related to Patient Engagement in Healthcare: Patient, Clinician and Researcher’s Perspectives. J Bioeth Inq. 2019;16(2):237–48. doi: 10.1007/s11673-019-09904-6 30741392

[49] Filliettaz SS, Laroussi-Libeault L, Rochat S, Desmedt SG. Lessons learned from the patient-as-partner approach in a postgraduate program in Switzerland. Santé Publique. 2024;36:57–68. doi: 10.3917/spub.243.0057 38906815

[50] Laroussi-Libeault L. Analyse réflexive du partenariat en santé à partir de mon expérience de patiente-partenaire: Constats, réflexions et recommandations. Partenariat soin patient analyses. 2022;Unlabeled volume. doi: 10.46298/pspa.14153

[51] Jacques B. Intégrer des patients experts, patients partenaires, patients formateurs ou cochercheurs en cancérologie : une démocratie en santé encore incertaine. Dialogue. 2022;61(1):33–53. doi: 10.1017/s0012217322000221

[52] Karazivan P, Dumez V, Flora L, Pomey M-P, Del Grande C, Ghadiri DP, et al. The patient-as-partner approach in health care: a conceptual framework for a necessary transition. Acad Med. 2015;90(4):437–41. doi: 10.1097/ACM.0000000000000603 25607943

[53] Laroussi-Libeault L, de Montgolfier S. Les enjeux éthiques du partenariat patient au service du système de santé : où en sommes nous avec les vulnérabilités ?.

[54] Ren M, Orsso CE, Ghomashchi H, da Silva BR, Aubrey C, Nielssen I, et al. Patient engagement strategies in digital health interventions for cancer survivors: A scoping review. PLOS Digit Health. 2025;4(5):e0000871. doi: 10.1371/journal.pdig.0000871 40446002 PMC12124549

[55] Goeldner M, Gehder S. Digital Health Applications (DiGAs) on a Fast Track: Insights From a Data-Driven Analysis of Prescribable Digital Therapeutics in Germany From 2020 to Mid-2024. J Med Internet Res. 2024;26:e59013. doi: 10.2196/59013 39208415 PMC11393499

