# Supplementary material for: Content and design considerations for digital supportive care tools for patients with cancer: A textual statistical analysis of patients’, relatives’, and healthcare professionals’ perspectives
Source: PLoS One. 2026 Oct 29;21(10):e0360665. doi: 10.1371/journal.pone.0360665 (PMC13718945; doi:10.1371/journal.pone.0360665)
Supplement: Supplementary file 1 [file pone.0360665.s001.docx]

| **Classe 1 (30.84%)** | | **Classe 2 (20.23%)** | | **Classe 3 (40.07%)** | | **Classe 4 (8.86%)** | |
| --- | --- | --- | --- | --- | --- | --- | --- |
| *Vécu du cancer* | | *Prise en soins des patient.e.s* | | *Imagination et positionnement dans la conceptualisation de l’outil : le contenu* | | *Forme de l’outil : le contenant* | |
| *Formes lexicales* | *χ²* | *Formes lexicales* | *χ²* | *Formes lexicales* | *χ²* | *Formes lexicales* | *χ²* |
| Traitement | 568,39 | Soin | 1753,85 | Information | 332,70 | Âgé | 737,97 |
| Cancer | 500,59 | Support | 1215,38 | Penser | 257,16 | Frein | 710,97 |
| Maladie | 347,66 | Charger | 481,26 | Chose | 239,22 | Âge | 567,58 |
| Chimio | 312,64 | Infirmier | 420,04 | Site | 208,35 | Téléphone | 504,45 |
| Mois | 298,20 | Psychologue | 360,40 | Outil | 164,62 | Ordinateur | 498,75 |
| Effet | 233,49 | Service | 360,20 | Question | 155,14 | Parole | 438,61 |
| Vivre | 190,60 | Equipe | 350,76 | Application | 153,96 | Informatique | 418,25 |
| Radiothérapie | 189,83 | Activité | 344,18 | Mettre | 143,67 | Groupe | 368,56 |
| Secondaire | 188,77 | Ville** | 262,93 | Trouver | 141,64 | Connecter | 325,78 |
| Sein | 174,36 | Palliatif | 251,13 | Intéressant | 123,81 | Tablette | 324,34 |
| Semaine | 139,72 | Hôpital | 249,36 | Chercher | 123,01 | Jeune | 267,75 |
| Femme | 128,49 | Centre | 214,65 | Répondre | 104,48 | Cohérence | 245,81 |
| Chimiothérapie | 126,60 | Ville | 202,40 | Internet | 104,44 | Génération | 218,04 |
| Normal | 124,55 | Médecin | 198,62 | Poser | 95,14 | Cardiaque | 218,04 |
| Moment | 122,23 | Médical | 182,15 | Besoin | 92,88 | Portable | 198,27 |
|  |  |  |  |  |  |  |  |
| *Absences significatives* | *χ²* | *Absences significatives* | *χ²* | *Absences significatives* | *χ²* | *Absences significatives* | *χ²* |
| Outil | -347,78 | Gens | -57,97 | Soin | -432,71 | Soin | -59,56 |
| Patient | -180,32 | Poser | -53,76 | Cancer | -215,03 | Information | -44,53 |
| Penser | -170,15 | Chose | -49,19 | Support | -212,13 | Traitement | -43,55 |
| Information | -169,99 | Question | -44,61 | Traitement | -202,88 | Prendre | -30,78 |
| Support | -134,63 | Téléphone | -42,00 | Charger | -139,54 | Question | -28,69 |
|  |  |  |  |  |  |  |  |
| *Formes supplémentaires* | *χ²* | *Formes supplémentaires* | *χ²* | *Formes supplémentaires* | *χ²* | *Formes supplémentaires* | *χ²* |
| Elle | 200,72 | Les | 76,51 | Pouvoir | 165,46 | Personne | 47,42 |
| Ma | 166,11 | De | 65,66 | Ça | 100,25 | Soixante-dix | 34,94 |
| Mon | 122,64 | Vers | 44,94 | Quelque | 72,85 | Soixante | 32,40 |
| Dire | 104,89 | Dans | 43,18 | Je | 72,78 | L | 25,93 |
| La | 89,86 | Par | 34,59 | Que | 68,77 | An | 21,97 |
|  |  |  |  |  |  |  |  |

**S1 Fig. Representation of the hierarchical descending classification of discourse analysis (Reinert’s analysis) in French.** Words grouping together all the cities mentioned by patients (replaced in the transcripts); All Chi² are significant at p < .001.

**S2 Fig. Similarity analysis of the DHC Class 1’s results with and without “to go” ("aller") in French.**

**S3 Fig. Similarity analysis of the DHC Class 2’s results in French.**

 **S4 Fig. Similarity analysis of the DHC Class 3’s results in French.**

**S5 Fig. Similarity analysis of the DHC Class 4’s results with and without tool ("outils") in French.**
