# Supplementary material for: Content and design considerations for digital supportive care tools for patients with cancer: A textual statistical analysis of patients’, relatives’, and healthcare professionals’ perspectives
Source: PLoS One. 2026 Oct 29;21(10):e0360665. doi: 10.1371/journal.pone.0360665 (PMC13718945; doi:10.1371/journal.pone.0360665)
Supplement: Peer review history

# Peer review: Decision Letter 0

**DOI:** 10.1371/journal.pone.0360665.r001

**Authors:** Ilhem Berrou

31 Aug 2026

Dear Dr. Zanni,

Thank you for submitting your manuscript to PLOS One. After careful consideration, we feel that it has merit but does not fully meet PLOS One’s publication criteria as it currently stands. Therefore, we invite you to submit a revised version of the manuscript that addresses the points raised during the review process.

Please submit your revised manuscript by Oct 15 2026 11:59PM. If you will need more time than this to complete your revisions, please reply to this message or contact the journal office at plosone@plos.org. When you're ready to submit your revision, log on to https://www.editorialmanager.com/pone/ and select the 'Submissions Needing Revision' folder to locate your manuscript file.

- A letter that responds to each point raised by the academic editor and reviewer(s). You should upload this letter as a separate file labeled 'Response to Reviewers'.
- A marked-up copy of your manuscript that highlights changes made to the original version. You should upload this as a separate file labeled 'Revised Manuscript with Track Changes'.
- An unmarked version of your revised paper without tracked changes. You should upload this as a separate file labeled 'Manuscript'.

If you would like to make changes to your financial disclosure, please include your updated statement in your cover letter. Guidelines for resubmitting your figure files are available below the reviewer comments at the end of this letter.

If applicable, we recommend that you deposit your laboratory protocols in protocols.io to enhance the reproducibility of your results. Protocols.io assigns your protocol its own identifier (DOI) so that it can be cited independently in the future. For instructions see: https://journals.plos.org/plosone/s/submission-guidelines#loc-laboratory-protocols. Additionally, PLOS One offers an option for publishing peer-reviewed Lab Protocol articles, which describe protocols hosted on protocols.io. Read more information on sharing protocols at https://plos.org/protocols?utm_medium=editorial-email&utm_source=authorletters&utm_campaign=protocols.

As the corresponding author, your ORCID iD is verified in the submission system and will appear in the published article. PLOS supports the use of ORCID, and we encourage all coauthors to register for an ORCID iD and use it as well. Please encourage your coauthors to verify their ORCID iD within the submission system before final acceptance, as unverified ORCID iDs will not appear in the published article. *Only*  the individual author can complete the verification step; PLOS staff *cannot*  verify ORCID iDs on behalf of authors.

We look forward to receiving your revised manuscript.

Kind regards,

Ilhem Berrou, PhD

Academic Editor

PLOS One

Journal Requirements:

When submitting your revision, we need you to address these additional requirements.

1. Please ensure that your manuscript meets PLOS ONE's style requirements, including those for file naming. The PLOS ONE style templates can be found at

https://journals.plos.org/plosone/s/file?id=wjVg/PLOSOne_formatting_sample_main_body.pdf and

https://journals.plos.org/plosone/s/file?id=ba62/PLOSOne_formatting_sample_title_authors_affiliations.pdf

2. In the ethics statement in the Methods, you have specified that verbal consent was obtained. Please provide additional details regarding how this consent was documented and witnessed, and state whether this was approved by the IRB.

3. We note that the grant information you provided in the ‘Funding Information’ and ‘Financial Disclosure’ sections do not match.

When you resubmit, please ensure that you provide the correct grant numbers for the awards you received for your study in the ‘Funding Information’ section.

4. Thank you for stating the following financial disclosure:

“This work was funded by the Ligue contre le cancer – Comité du Nord (North France Cancer League) and the French National Cancer Institute (grant INCA/16136) in partnership with the University of Lille, the SCALab laboratory (UMR CNRS 9193), the ONCOLille Institute, and the Oscar Lambret Center, which support the Opsyrii research chair “innovations in psycho-oncology and interventional research.”

Please state what role the funders took in the study. If the funders had no role, please state: "The funders had no role in study design, data collection and analysis, decision to publish, or preparation of the manuscript."

If this statement is not correct you must amend it as needed.

Please include this amended Role of Funder statement in your cover letter; we will change the online submission form on your behalf.

5. Thank you for stating the following in your Competing Interests section:

“The authors declare no competing interests. The authors have no relevant financial or non-financial interests to disclose.”

Please complete your Competing Interests on the online submission form to state any Competing Interests. If you have no competing interests, please state "The authors have declared that no competing interests exist.", as detailed online in our guide for authors at http://journals.plos.org/plosone/s/submit-now

This information should be included in your cover letter; we will change the online submission form on your behalf.

6. We note that you have indicated that there are restrictions to data sharing for this study. For studies involving human research participant data or other sensitive data, we encourage authors to share de-identified or anonymized data. However, when data cannot be publicly shared for ethical reasons, we allow authors to make their data sets available upon request. For information on unacceptable data access restrictions, please see http://journals.plos.org/plosone/s/data-availability#loc-unacceptable-data-access-restrictions.

Before we proceed with your manuscript, please address the following prompts:

a) If there are ethical or legal restrictions on sharing a de-identified data set, please explain them in detail (e.g., data contain potentially identifying or sensitive patient information, data are owned by a third-party organization, etc.) and who has imposed them (e.g., a Research Ethics Committee or Institutional Review Board, etc.). Please also provide contact information for a data access committee, ethics committee, or other institutional body to which data requests may be sent.

b) If there are no restrictions, please upload the minimal anonymized data set necessary to replicate your study findings to a stable, public repository and provide us with the relevant URLs, DOIs, or accession numbers. Please see http://www.bmj.com/content/340/bmj.c181.long for guidelines on how to de-identify and prepare clinical data for publication. For a list of recommended repositories, please see https://journals.plos.org/plosone/s/recommended-repositories. You also have the option of uploading the data as Supporting Information files, but we would recommend depositing data directly to a data repository if possible.

Please update your Data Availability statement in the submission form accordingly.

7. We note that you have referenced (ie. Bewick et al. [5]) which has currently not yet been accepted for publication. Please remove this from your References and amend this to state in the body of your manuscript: (ie “Bewick et al. [Unpublished]”) as detailed online in our guide for authors

http://journals.plos.org/plosone/s/submission-guidelines#loc-reference-style

8. If the reviewer comments include a recommendation to cite specific previously published works, please review and evaluate these publications to determine whether they are relevant and should be cited. There is no requirement to cite these works unless the editor has indicated otherwise.

9. Please review your reference list to ensure that it is complete and correct. If you have cited papers that have been retracted, please include the rationale for doing so in the manuscript text, or remove these references and replace them with relevant current references. Any changes to the reference list should be mentioned in the rebuttal letter that accompanies your revised manuscript. If you need to cite a retracted article, indicate the article’s retracted status in the References list and also include a citation and full reference for the retraction notice.

[Note: HTML markup is below. Please do not edit.]

Reviewers' comments:

Reviewer's Responses to Questions

**Comments to the Author**

1. Is the manuscript technically sound, and do the data support the conclusions?

Reviewer #1: Yes

Reviewer #2: Yes

**********

2. Has the statistical analysis been performed appropriately and rigorously?

Reviewer #1: Yes

Reviewer #2: I Don't Know

**********

3. Have the authors made all data underlying the findings in their manuscript fully available?

The PLOS Data policy

Reviewer #1: Yes

Reviewer #2: Yes

**********

4. Is the manuscript presented in an intelligible fashion and written in standard English?

Reviewer #1: Yes

Reviewer #2: Yes

**********

Reviewer #1: Abstract

The abstract lists four classes that emerged from the analysis however, the subsequent statement refers to "across the three groups." Please confirm whether this is an error and revise as appropriate.

Introduction

The background is well presented, and the rationale for the study is clearly articulated. The study aim is also clearly stated. Well done.

Methods

1. Please ensure that references are cited consistently throughout the manuscript (i.e., OSF reference).

2. I recommend moving the paragraph in lines 185-189, which provides the rationale for the textual statistical analysis, to immediately follow the statement in lines 120–121: “The initial analysis involved a thematic analysis [17] of participants’ accounts, conducted separately for each group [18–20].” Presenting the rationale immediately after the description of the initial thematic analysis would improve the logical flow of the methods section by introducing the motivation for the subsequent secondary analysis before describing the procedures.

3. The description of the final stage of the analysis would benefit from additional detail to improve transparency. In particular, it would be helpful to clarify what is meant by "qualitative inductive analyses according to the quantitative outputs were conducted" and how the quantitative findings informed the qualitative interpretation. For example, were the quantitative outputs used to guide the coding framework, interpretation, or the selection of text segments for further analysis?

4. Please provide a little more information on how the independent analyses conducted by MR and LZ were combined. Rather than stating that the analyses were merged and that discussions were held until a compromise was reached, it would be helpful to briefly describe how differing interpretations were discussed and refined to arrive at the final analysis. For example, were disagreements resolved solely through discussion between MR and LZ, or were interpretations reviewed with an additional qualitative researcher or the broader research team?

Discussion

1. The authors have done a good job addressing the first study aim of documenting and comparing the perspectives of the different participant groups. However, the second aim (to identify barriers and facilitators) receives comparatively less explicit attention in the discussion. Although barriers and facilitators are described throughout the manuscript, they are not synthesised or discussed as directly in relation to the stated aim. I encourage the authors to more explicitly discuss the key barriers and facilitators identified by the analysis and their implications. This would ensure that both study aims receive equal emphasis and help readers more clearly appreciate how the findings address the objectives of the study.

2. I commend the authors for drawing on both UTAUT and CFIR to interpret the findings. Integrating a technology acceptance framework with an implementation science framework strengthens the discussion and provides a richer understanding of the factors influencing digital health adoption.

However, the statement in lines 551-552 is unclear: "UTAUT is embedded within a broader implementation framework, the Consolidated Framework for Implementation Research." It would be helpful to clarify what is meant by "embedded." Did the authors integrate UTAUT within CFIR to understand how technology acceptance interacts with implementation context, intervention characteristics, and implementation processes? If so, was this analytical approach adapted from previous literature, or is it a novel contribution of this study? Either way, this is a good approach, but the manuscript would benefit from a clearer explanation of why the two frameworks were combined and the new insight this combination offers.

Limitations

In the limitations section, the authors state that having different individuals conduct the interviews and the analyses was a limitation of the study. I am not convinced that this is necessarily a limitation. It is common practice in qualitative research for data collection and analysis to be undertaken by different members of the research team. If appropriate procedures were in place to preserve contextual understanding of the interviews (e.g., detailed notes and debriefings), this approach can be entirely appropriate. I encourage the authors to either provide further justification for why this constituted a limitation in the context of their study or reconsider its inclusion as a study limitation.

Reviewer #2: Please review and clarify lines 322-324: 322 “Needs change as the disease progresses, and they are not necessarily the same 323 depending on the stage of treatment. I have a friend who has been undergoing intensive

324 treatment, including chemotherapy, for almost two years.” (Pauline, 30 years old, patient)' is this a patient perspective or of a carer i.e. is the information in the parentheses correct? Please review and confirm as appropriate.

**********

what does this mean?). If published, this will include your full peer review and any attached files.

If you choose “no”, your identity will remain anonymous but your review may still be made public.

**Do you want your identity to be public for this peer review?**  For information about this choice, including consent withdrawal, please see our Privacy Policy

Reviewer #1: No

Reviewer #2: No

**********

[NOTE: If reviewer comments were submitted as an attachment file, they will be attached to this email and accessible via the submission site. Please log into your account, locate the manuscript record, and check for the action link "View Attachments". If this link does not appear, there are no attachment files.]

To ensure your figures meet our technical requirements, please review our figure guidelines: https://journals.plos.org/plosone/s/figures

You may also use PLOS’s free figure tool, NAAS, to help you prepare publication quality figures: https://journals.plos.org/plosone/s/figures#loc-tools-for-figure-preparation.

NAAS will assess whether your figures meet our technical requirements by comparing each figure against our figure specifications.

# Author comment: Author response to Decision Letter 1

**DOI:** 10.1371/journal.pone.0360665.r002

28 Sep 2026

General reply

We would like to thank the editor and reviewers for their interesting initial reviews, which have helped us to advance our thinking and the quality of this manuscript. We have tried to respond as appropriately as possible to all the comments formulated.

We are therefore returning three documents:

- a point-by-point response to each reviewer,

- a marked-up version of the manuscript (modifications in red, purple or green, depending on who we were replying to),

- a clean version of the manuscript.

We have also amended Figures 2, 3, 4 and 5 to improve their visual quality.

Response at Editor – changes will be highlighted in red in the annotated document

2. In the ethics statement in the Methods, you have specified that verbal consent was obtained. Please provide additional details regarding how this consent was documented and witnessed, and state whether this was approved by the IRB.

Response: We have added the following information:

Line 125-126: “As required by law and reported to the ethics committee, oral consent was obtained from all participants upon inclusion and audio-recorded before the research interview began.”

3. We note that the grant information you provided in the ‘Funding Information’ and ‘Financial Disclosure’ sections do not match. When you resubmit, please ensure that you provide the correct grant numbers for the awards you received for your study in the ‘Funding Information’ section.

Response: Please accept our apologies for this mistake. The information was corrected.

4. Thank you for stating the following financial disclosure:

“This work was funded by the Ligue contre le cancer – Comité du Nord (North France Cancer League) and the French National Cancer Institute (grant INCA/16136) in partnership with the University of Lille, the SCALab laboratory (UMR CNRS 9193), the ONCOLille Institute, and the Oscar Lambret Center, which support the Opsyrii research chair “innovations in psycho-oncology and interventional research.”

Please state what role the funders took in the study. If the funders had no role, please state: "The funders had no role in study design, data collection and analysis, decision to publish, or preparation of the manuscript."

If this statement is not correct you must amend it as needed.

Please include this amended Role of Funder statement in your cover letter; we will change the online submission form on your behalf.

Response: The funders had no role in the study. Hence, we added the recommended sentence.

5. Thank you for stating the following in your Competing Interests section:

“The authors declare no competing interests. The authors have no relevant financial or non-financial interests to disclose.”

Please complete your Competing Interests on the online submission form to state any Competing Interests. If you have no competing interests, please state "The authors have declared that no competing interests exist.", as detailed online in our guide for authors at http://journals.plos.org/plosone/s/submit-now

This information should be included in your cover letter; we will change the online submission form on your behalf.

Response: We have changed our sentence in Competing interest.

6. We note that you have indicated that there are restrictions to data sharing for this study. For studies involving human research participant data or other sensitive data, we encourage authors to share de-identified or anonymized data. However, when data cannot be publicly shared for ethical reasons, we allow authors to make their data sets available upon request. For information on unacceptable data access restrictions, please see http://journals.plos.org/plosone/s/data-availability#loc-unacceptable-data-access-restrictions.

a) If there are ethical or legal restrictions on sharing a de-identified data set, please explain them in detail (e.g., data contain potentially identifying or sensitive patient information, data are owned by a third-party organization, etc.) and who has imposed them (e.g., a Research Ethics Committee or Institutional Review Board, etc.). Please also provide contact information for a data access committee, ethics committee, or other institutional body to which data requests may be sent.

b) If there are no restrictions, please upload the minimal anonymized data set necessary to replicate your study findings to a stable, public repository and provide us with the relevant URLs, DOIs, or accession numbers. Please see http://www.bmj.com/content/340/bmj.c181.long for guidelines on how to de-identify and prepare clinical data for publication. For a list of recommended repositories, please see https://journals.plos.org/plosone/s/recommended-repositories. You also have the option of uploading the data as Supporting Information files, but we would recommend depositing data directly to a data repository if possible.

Please update your Data Availability statement in the submission form accordingly.

Response: Our data cannot be shared as it contains potentially identifying or sensitive patient information, as recommended by the ethics approvals obtained to conduct the study. However, we added the following sentence with contact information:

“However, the interviews cannot be shared as they contain potentially identifying or sensitive patient information. Information related to data can be addressed to: https://scalab.univ-lille.fr/acces-contact”

7. We note that you have referenced (ie. Bewick et al. [5]) which has currently not yet been accepted for publication. Please remove this from your References and amend this to state in the body of your manuscript: (ie “Bewick et al. [Unpublished]”) as detailed online in our guide for authors

http://journals.plos.org/plosone/s/submission-guidelines#loc-reference-style

Response: All the references that had not been published have now been published; we have rectified this.

9. Please review your reference list to ensure that it is complete and correct. If you have cited papers that have been retracted, please include the rationale for doing so in the manuscript text, or remove these references and replace them with relevant current references. Any changes to the reference list should be mentioned in the rebuttal letter that accompanies your revised manuscript. If you need to cite a retracted article, indicate the article’s retracted status in the References list and also include a citation and full reference for the retraction notice.

Response: We reviewed our reference list and haven’t retracted paper to our knowledge. Thank you for your attention.

Response at Reviewer #1 – changes will be highlighted in violet in the annotated document

Abstract

The abstract lists four classes that emerged from the analysis however, the subsequent statement refers to "across the three groups." Please confirm whether this is an error and revise as appropriate.

Response: Please accept our apologies for this unclear sentence. The three groups correspond to patients, relatives and healthcare professionals. We have added a few words to clarify the abstract.

Line 42: “three different groups ”.

Introduction

The background is well presented, and the rationale for the study is clearly articulated. The study aim is also clearly stated. Well done.

Response: Thank you!

Methods

1. Please ensure that references are cited consistently throughout the manuscript (i.e., OSF reference).

Response: Thank you. We have checked this (see reply to the editor above).

2. I recommend moving the paragraph in lines 185-189, which provides the rationale for the textual statistical analysis, to immediately follow the statement in lines 120–121: “The initial analysis involved a thematic analysis [17] of participants’ accounts, conducted separately for each group [18–20].” Presenting the rationale immediately after the description of the initial thematic analysis would improve the logical flow of the methods section by introducing the motivation for the subsequent secondary analysis before describing the procedures.

Response: Thank you for this suggestion. We have acted on it and moved this paragraph.

3. The description of the final stage of the analysis would benefit from additional detail to improve transparency. In particular, it would be helpful to clarify what is meant by "qualitative inductive analyses according to the quantitative outputs were conducted" and how the quantitative findings informed the qualitative interpretation. For example, were the quantitative outputs used to guide the coding framework, interpretation, or the selection of text segments for further analysis?

Response: Thank you for your feedback: you’re right, what we thought was self-evident – as we’d done it ourselves – wasn’t explained clearly enough. We have therefore added:

Line 225-229: “Qualitative analysis involved interpreting the quantitative output (χ² and figures): descriptions of the active, supplementary forms and associated verbatim of each class and subclass, inferences about their meaning, examination of the links between central and peripheral words in the similarity graphs, and explanation of the links to the associated variables.”

4. Please provide a little more information on how the independent analyses conducted by MR and LZ were combined. Rather than stating that the analyses were merged and that discussions were held until a compromise was reached, it would be helpful to briefly describe how differing interpretations were discussed and refined to arrive at the final analysis. For example, were disagreements resolved solely through discussion between MR and LZ, or were interpretations reviewed with an additional qualitative researcher or the broader research team?

Response: Additional information has been provided to clarify this point in the manuscript:

Line 229-233: “Discussions were held on the results until a compromise was reached. The two independent analyses were combined by reviewing each category and comparing interpretations. Any difficulties or disagreements were noted for inclusion in the results; a discussion session with other researchers familiar with the interviews (AN and KL) was planned in the event of persistent disagreements, should the need arise, but this proved unnecessary.”

Discussion

1. The authors have done a good job addressing the first study aim of documenting and comparing the perspectives of the different participant groups.

Response: Thank you for your feedback.

However, the second aim (to identify barriers and facilitators) receives comparatively less explicit attention in the discussion. Although barriers and facilitators are described throughout the manuscript, they are not synthesised or discussed as directly in relation to the stated aim. I encourage the authors to more explicitly discuss the key barriers and facilitators identified by the analysis and their implications. This would ensure that both study aims receive equal emphasis and help readers more clearly appreciate how the findings address the objectives of the study.

Response: We understand your comment. Indeed, the link to our second objective was unclear. However, on reviewing our paragraphs, we noticed that the barriers and facilitators were implied. We have therefore added a sentence to summarize all of this between the discussion of the results and the link to UTAUT, which we revisit below:

Line 552-555: “Taken together, these findings address the study's second aim by identifying key barriers (limited digital literacy linked to the age, concerns about data protection) and facilitators, including ease of access, personalization, and the preservation of human mediation through HCP follow-up.”

2. I commend the authors for drawing on both UTAUT and CFIR to interpret the findings. Integrating a technology acceptance framework with an implementation science framework strengthens the discussion and provides a richer understanding of the factors influencing digital health adoption.

However, the statement in lines 551-552 is unclear: "UTAUT is embedded within a broader implementation framework, the Consolidated Framework for Implementation Research." It would be helpful to clarify what is meant by "embedded." Did the authors integrate UTAUT within CFIR to understand how technology acceptance interacts with implementation context, intervention characteristics, and implementation processes? If so, was this analytical approach adapted from previous literature, or is it a novel contribution of this study? Either way, this is a good approach, but the manuscript would benefit from a clearer explanation of why the two frameworks were combined and the new insight this combination offers.

Response: Thank you for your feedback! We did indeed want to incorporate UTAUT into the CFIR framework, so that we could use the points highlighted in the first model to implement the digital tools. Hence, we modified the paragraph in the discussion to add more details:

“Within the Unified Theory of Acceptance and Use of Technology (UTAUT) model [34], particularly the extension of this model by Schretzlmaier et al. [35], the use of digital tools is influenced by habits and facilitating conditions. This perspective is reflected in participants’ comments, which emphasise the importance of easy access to information and an appropriate, user-friendly interface. These acceptance factors highlighted in UTAUT can be situated within a broader implementation framework, in particular through UTAUT is embedded within a broader implementation framework, the Consolidated Framework for Implementation Research (CFIR; [36]), which highlights the need to leverage characteristics of the intervention itself, such as avoiding complexity, to support implementation. Beyond intervention characteristics, the CFIR identifies four domains that may influence implementation, including external factors (e.g. patient needs and available resources, which were explored in this study and align with UTAUT's facilitating conditions), the characteristics of the inner setting (notably, organisational norms and values, including support from patients and relatives partners, a point to which we return below), individual characteristics (which map onto UTAUT's habits and acceptance factors), and the implementation process itself. Although not all of these domains were addressed here, combining UTAUT with CFIR this framework provides a useful lens for understanding how design, context, and process interact in shaping the uptake of digital health tools. “

Limitations

In the limitations section, the authors state that having different individuals conduct the interviews and the analyses was a limitation of the study. I am not convinced that this is necessarily a limitation. It is common practice in qualitative research for data collection and analysis to be undertaken by different members of the research team. If appropriate procedures were in place to preserve contextual understanding of the interviews (e.g., detailed notes and debriefings), this approach can be entirely appropriate. I encourage the authors to either provide further justification for why this constituted a limitation in the context of their study or reconsider its inclusion as a study limitation.

Response: We have given your suggestion further thought, and we do indeed agree. We have removed this part from our limitations.

Response at Reviewer #2 – changes will be highlighted in green in the annotated document

Please review and clarify lines 322-324: 322 “Needs change as the disease progresses, and they are not necessarily the same 323 depending on the stage of treatment. I have a friend who has been undergoing intensive 324 treatment, including chemotherapy, for almost two years.” (Pauline, 30 years old, patient)' is this a patient perspective or of a carer i.e. is the information in the parentheses correct? Please review and confirm as appropriate.

Response: Thank you for your feedback. It is, in fact, a patient speaking about another patient – as we made clear in the text:

Line 317-318 : « [talking about another patient] ».

Attachment Submitted filename: Response to Reviewers.docx

# Editor report: Decision Letter 1

**DOI:** 10.1371/journal.pone.0360665.r003

**Authors:** Ilhem Berrou

29 Sep 2026

Content and design considerations for digital supportive care tools for patients with cancer: A textual statistical analysis of patients’, relatives’, and healthcare professionals’ perspectives.

PONE-D-26-10110R1

Dear Dr. Zanni,

We’re pleased to inform you that your manuscript has been judged scientifically suitable for publication and will be formally accepted for publication once it meets all outstanding technical requirements.

Within one week, you’ll receive an e-mail detailing the required amendments. When these have been addressed, you’ll receive a formal acceptance letter and your manuscript will be scheduled for publication.

An invoice will be generated when your article is formally accepted. Please note, if your institution has a publishing partnership with PLOS and your article meets the relevant criteria, all or part of your publication costs will be covered. Please make sure your user information is up-to-date by logging into Editorial Manager at Editorial Manager® and clicking the ‘Update My Information' link at the top of the page. For questions related to billing, please contact billing support.

If your institution or institutions have a press office, please notify them about your upcoming paper to help maximize its impact. If they’ll be preparing press materials, please inform our press team as soon as possible -- no later than 48 hours after receiving the formal acceptance. Your manuscript will remain under strict press embargo until 2 pm Eastern Time on the date of publication. For more information, please contact onepress@plos.org.

Kind regards,

Ilhem Berrou, PhD

Academic Editor

PLOS One

Additional Editor Comments (optional):

Please note the repeat of sentence at the end of page 6

# Editor report: Acceptance letter

**DOI:** 10.1371/journal.pone.0360665.r004

**Authors:** Ilhem Berrou

PONE-D-26-10110R1

PLOS One

Dear Dr. Zanni,

I'm pleased to inform you that your manuscript has been deemed suitable for publication in PLOS One. Congratulations! Your manuscript is now being handed over to our production team.

At this stage, our production department will prepare your paper for publication. This includes ensuring the following:

* All references, tables, and figures are properly cited

* All relevant supporting information is included in the manuscript submission,

* There are no issues that prevent the paper from being properly typeset

You will receive further instructions from the production team, including instructions on how to review your proof when it is ready. Please keep in mind that we are working through a large volume of accepted articles, so please give us a few days to review your paper and let you know the next and final steps.

Lastly, if your institution or institutions have a press office, please let them know about your upcoming paper now to help maximize its impact. If they'll be preparing press materials, please inform our press team within the next 48 hours. Your manuscript will remain under strict press embargo until 2 pm Eastern Time on the date of publication. For more information, please contact onepress@plos.org.

You will receive an invoice from PLOS for your publication fee after your manuscript has reached the completed accept phase. If you receive an email requesting payment before acceptance or for any other service, this may be a phishing scheme. Learn how to identify phishing emails and protect your accounts at https://explore.plos.org/phishing.

If we can help with anything else, please email us at customercare@plos.org.

Thank you for submitting your work to PLOS One and supporting open access.

Kind regards,

PLOS One Editorial Office Staff

on behalf of

Dr. Ilhem Berrou

Academic Editor

PLOS One
